# Cross linguistic cognitive load estimation via hierarchical attention in English translation education

**DOI:** 10.3389/fpsyg.2026.1771134

**Published:** 2026-06-17

**Authors:** Xiaoyan Dong

**Affiliations:** Yancheng Institute of Technology, Yancheng, Jiangsu, China

**Keywords:** bayesian cognitive load refinement, cross linguistic cognitive load, english translation education, hierarchical attention mechanism, variational constraint optimization

## Abstract

**Introduction:**

Estimating cognitive load in English translation education is important for understanding learners' processing difficulty and supporting adaptive instructional decisions. However, cognitive load is a latent psychological construct and cannot be treated as a directly observed token level label. To address this issue, this study proposes a cross linguistic cognitive load estimation framework based on hierarchical attention and probabilistic modeling.

**Methods:**

Cognitive load proxy scores are constructed from human derived translation process measurements, including subjective workload ratings, eye tracking, pupillometry, keystroke logs, timing records, and translation performance indicators. The proposed Hierarchical Cognitive Load Estimator integrates three modules: a Variational Constraint Optimizer, an Attention guided Temporal Segmenter, and an Uncertainty aware Output Regularizer. These modules enable the model to capture token , segment , and document level dependencies, represent latent learner and task specific factors, and estimate predictive uncertainty. The task is formulated as continuous probabilistic regression rather than categorical classification. Experiments are conducted on translation process datasets covering English-Chinese, English-Japanese, and English-Spanish settings, with standard, cross subject, and cross language evaluation protocols. The proposed model is compared with feature based regression, neural sequence models, structured state space models, hierarchical attention baselines, and probabilistic uncertainty baselines.

**Results and discussion:**

Results show that the proposed framework improves regression performance, probabilistic fit, and uncertainty calibration. Additional ablation, sensitivity, interpretability, and educator facing analyzes indicate that the model's predictions align with known difficulty indicators and can support instructional diagnosis, learner feedback, curriculum adjustment, and adaptive intervention in translation education.

## 1 Introduction

The task of cross linguistic cognitive load estimation in English translation education is of paramount importance due to its potential to enhance the learning experience and improve translation quality. Not only does it provide insights into the cognitive challenges faced by learners during translation tasks, but it also enables the development of adaptive teaching strategies tailored to individual needs (Yang et al., 2016). Furthermore, understanding cognitive load can contribute to the design of more effective educational tools and resources, fostering better comprehension and retention of linguistic knowledge (Leppink et al., 2013). This task is particularly significant in the context of English translation education, where learners often grapple with complex syntactic structures, cultural nuances, and semantic ambiguities (Dragsted, 2005). By accurately estimating cognitive load, educators can identify bottlenecks in the learning process and implement targeted interventions, ultimately bridging the gap between theoretical knowledge and practical application. Therefore, research in this domain is not only academically intriguing but also practically impactful, addressing the pressing need for personalized and efficient language education methodologies.

Initial efforts to estimate cognitive load in translation education focused on manually crafted systems that relied on predefined rules and expert knowledge. These approaches aimed to model linguistic structures and translation processes explicitly, capturing the cognitive demands of translation tasks through structured frameworks (Carl and Kay, 2011). However, the reliance on handcrafted features and domain specific knowledge posed significant challenges, particularly in terms of scalability and adaptability to diverse linguistic contexts (Schaeffer and Carl, 2013). Moreover, these methods often resulted in oversimplified models that struggled to account for the dynamic and context dependent nature of cognitive load (Alves and Albir, 2017). Despite these limitations, these early systems laid the groundwork for subsequent advancements by highlighting the importance of structured knowledge and interpretability in cognitive load estimation.

As the field evolved, researchers began to explore more flexible approaches that could adapt to varying linguistic and educational contexts. By employing statistical models and leveraging large scale datasets, these methods sought to infer patterns and relationships between linguistic features and cognitive load (Xiao and Mart́ın, 2020). Techniques such as regression models and support vector machines were utilized to predict cognitive load based on features like sentence length, word frequency, and syntactic complexity (Alves and Jakobsen, 2021). While these approaches demonstrated improved performance and generalizability, they were often limited by their dependence on feature engineering and the quality of annotated datasets. The interpretability of these models posed a challenge, as their predictions were frequently difficult to explain in terms of the underlying cognitive processes. Nevertheless, these methods represented a crucial step forward, paving the way for more sophisticated techniques in cognitive load estimation.

In recent years, the introduction of deep learning and pre trained models has transformed cognitive load estimation, offering unprecedented accuracy and scalability. These methods utilize neural networks and large scale pre trained language models to capture complex linguistic patterns and contextual information. By employing hierarchical attention mechanisms and multi layer architectures, deep learning models effectively model the hierarchical and context dependent nature of cognitive load in translation tasks (O'brien, 2006). Furthermore, transfer learning and fine tuning techniques enable these models to adapt to specific educational settings and linguistic domains, enhancing their applicability and robustness. Despite their remarkable performance, deep learning methods are not without limitations. They often require substantial computational resources and large annotated datasets, which can be challenging to obtain in educational contexts. Their black box nature raises concerns about interpretability and transparency, complicating efforts to understand the underlying mechanisms driving their predictions. Nonetheless, deep learning and pre trained models represent the state of the art in cognitive load estimation, offering a powerful foundation for future research and applications.

Based on the aforementioned limitations of traditional, data driven, and deep learning approaches, we propose a novel method for cross linguistic cognitive load estimation in English translation education. Our approach addresses the scalability and adaptability challenges of symbolic AI, the feature engineering and interpretability issues of data driven methods, and the computational and transparency concerns of deep learning models. By integrating hierarchical attention mechanisms with domain specific linguistic features, our method combines the strengths of structured knowledge representation and data driven learning. This hybrid approach not only enhances the accuracy and robustness of cognitive load estimation but also ensures interpretability and scalability across diverse educational contexts. Furthermore, our method leverages transfer learning and fine tuning techniques to adapt to specific linguistic and cultural nuances, enabling personalized and context aware cognitive load estimation. Through extensive experiments and evaluations, we demonstrate the effectiveness of our approach in addressing the unique challenges of English translation education, paving the way for more efficient and personalized language learning methodologies.

We summarize our contributions as follows:

- We propose a hierarchical attention based approach that integrates domain specific linguistic features, enabling accurate, and interpretable cognitive load estimation.
- Our method demonstrates high scalability and adaptability, making it suitable for diverse educational contexts and linguistic domains.
- Experimental results show improved regression performance, uncertainty calibration, and interpretability compared with feature based, neural sequence, hierarchical attention, and probabilistic baseline models.
- We further articulate how continuous cognitive load estimates can be translated into educational actions, including instructional diagnosis, learner feedback, curriculum adjustment, and adaptive intervention in English translation education.

## 2 Related work

### 2.1 Cognitive load theory and translation education

Cognitive load theory provides the primary theoretical foundation for the present study. The theory assumes that human working memory has limited capacity and that learning outcomes are strongly influenced by how instructional materials distribute cognitive demands across intrinsic, extraneous, and germane load (Sweller, 1988). Intrinsic load is associated with the inherent complexity of the learning material, extraneous load is imposed by suboptimal instructional design, and germane load reflects cognitive resources devoted to schema construction and meaningful learning (Sweller et al., 1998). In translation education, these three forms of load are particularly relevant because learners must simultaneously comprehend the source text, maintain cross linguistic correspondences in working memory, resolve lexical and syntactic ambiguities, and produce an acceptable target language output (Paas et al., 2016). Translation tasks can impose high intrinsic load when the source text contains dense syntactic structures, low frequency vocabulary, idiomatic expressions, or culturally specific references. They can also impose extraneous load when learners receive insufficient task scaffolding, unclear feedback, or poorly sequenced translation materials. From an instructional perspective, estimating cognitive load is therefore not merely a prediction problem but also a way to identify when a translation task exceeds the learner's available cognitive resources. This perspective is consistent with educational psychology research showing that cognitive load measures can guide task sequencing, instructional support, and adaptive learning interventions (Van Merrienboer and Sweller, 2005). In the context of English translation education, cognitive load estimation can support the design of graded translation exercises, individualized feedback, and timely intervention. For example, if a model detects high load associated with syntactic reordering or lexical ambiguity, the teacher may provide targeted scaffolds such as parallel examples, segmentation prompts, or contrastive analysis. Accordingly, the present study treats cognitive load as an educationally meaningful latent construct rather than as a purely computational output.

### 2.2 Translation process research and cognitive load measurement

Translation process research has developed a rich set of empirical methods for studying the cognitive effort involved in translation. Eye tracking studies have shown that fixation duration, gaze duration, regressions, and pupil dilation can reflect processing difficulty during reading and translation (Paas, 1992). Keystroke logging and pause analysis provide complementary behavioral evidence by capturing production effort, revision behavior, hesitation, and problem solving episodes during translation (Hart and Staveland, 1988). These measures are particularly useful because translation is a temporally extended task in which comprehension and production processes interact dynamically. Subjective workload instruments have also been widely used to assess perceived task difficulty and mental effort. The Paas mental effort rating scale provides a concise measure of perceived cognitive effort during learning tasks, while the NASA TLX measures workload across multiple dimensions, including mental demand, temporal demand, effort, and frustration (Jakobsen, 2002). In translation education, such subjective measures can be combined with process data, such as gaze behavior, response time, revision frequency, and keystroke pauses, to obtain a more reliable estimate of learners' cognitive load (Sharon, 2007). These traditions suggest that cognitive load should be operationalized through converging evidence from self report, behavioral, and physiological indicators. Therefore, the present study does not conceptualize token level load as an independently observed psychological state. Rather, token, segment , and document level load values are treated as aligned estimates derived from multiple measurement channels. This distinction is important because cognitive load is a latent construct, and its computational modeling must be grounded in transparent measurement and validation procedures.

### 2.3 Cross linguistic processing and hierarchical computational modeling

Cross linguistic translation involves multiple levels of processing, including lexical access, syntactic restructuring, semantic integration, pragmatic interpretation, and target language production. Psycholinguistic studies on bilingual processing have shown that two languages may be co activated during comprehension and production, and that cross linguistic transfer can influence processing difficulty (Carl, 2012). In translation tasks, learners often need to inhibit direct word for word transfer, reorganize sentence structures, and resolve mismatches between source language and target language conventions. These operations can increase cognitive load, especially for novice translators. A hierarchical modeling perspective is well suited to this problem because translation difficulty is not located only at the word level. Local lexical difficulty, phrase level ambiguity, sentence level syntactic complexity, and document level discourse coherence may jointly shape cognitive effort. Hierarchical attention mechanisms can therefore be used as computational tools to aggregate evidence across tokens, segments, and whole translation units. In the present study, attention is not used as a substitute for psychological theory. Instead, it is used to represent the multi level structure already emphasized by cognitive load theory and translation process research (Hvelplund, 2014). Recent educational data modeling studies have increasingly combined neural sequence models with interpretable features and uncertainty estimation to support adaptive learning. For translation education, such models are valuable only when their outputs can be connected to theoretically meaningful indicators, such as dependency length, lexical frequency, syntactic complexity, gaze duration, pupil dilation, pause duration, and subjective mental effort. The proposed framework follows this principle by integrating hierarchical linguistic representations with cognitive load measurements and uncertainty aware prediction. This design aims to bridge computational modeling and educational interpretation, enabling the model to support not only prediction accuracy but also pedagogically actionable feedback.

## 3 Method

### 3.1 Overview

This section delineates the methodological framework devised for Cross Linguistic Cognitive Load Estimation through Hierarchical Attention within the realm of English Translation Education. The study's primary aim is to tackle the complexities inherent in estimating cognitive load across linguistic boundaries, in the context of English translation pedagogy. To this end, we propose an innovative model, the *Hierarchical Cognitive Load Estimator*, which employs hierarchical attention mechanisms to elucidate the intricate interactions between linguistic attributes and cognitive load. The methodology is systematically divided into three principal components: *Preliminaries, Hierarchical Cognitive Load Estimator*, and *Bayesian Cognitive Load Refinement*.

In Section 3.2, foundational concepts are established, and the problem of cross linguistic cognitive load estimation is formalized. This involves articulating the mathematical framework and notations essential for representing the interplay between linguistic features, translation tasks, and cognitive load. The preliminaries section also introduces the hierarchical structure of attention mechanisms, which constitute the core of the proposed model. By formalizing the problem, we aim to provide a precise and rigorous foundation for the subsequent development of the model and strategy.

Section 3.3 presents the *Hierarchical Cognitive Load Estimator*, a novel model crafted to address the intricacies of cognitive load estimation in cross linguistic scenarios. The model integrates three distinct modules: the *Variational Constraint Optimizer*, the *Attention guided Temporal Segmenter*, and the *Uncertainty aware Output Regularizer*. Each module is meticulously engineered to tackle specific facets of the problem, such as optimizing constraints, segmenting temporal data, and managing uncertainty in predictions. The hierarchical attention mechanism embedded within the model facilitates the integration of multi level linguistic features, enabling a nuanced comprehension of cognitive load dynamics.

In Section 3.4, the *Bayesian Cognitive Load Refinement* strategy is elaborated, complementing the *Hierarchical Cognitive Load Estimator* by refining the optimization process and addressing uncertainty in the model's outputs. This strategy is founded on two principles: variational regularization and uncertainty aware probabilistic prediction. Variational regularization constrains the latent representation so that it remains stable and consistent with the hierarchical structure of cognitive load estimation. Uncertainty aware probabilistic prediction models both the expected cognitive load proxy and the associated predictive variance, thereby reducing overconfident estimates in ambiguous translation cases.

Collectively, these sections constitute a cohesive methodological framework that confronts the challenges of cross linguistic cognitive load estimation. By integrating hierarchical attention mechanisms, advanced optimization techniques, and uncertainty aware strategies, the proposed approach offers a novel resolution to the problem, with profound implications for English translation education. The ensuing sections delve into the technical intricacies of each component, providing a rigorous and systematic exposition of the methodology.

### 3.2 Preliminaries

This subsection formalizes the problem of cross linguistic cognitive load estimation within the context of English translation education. The objective is to establish a mathematical framework that captures the hierarchical and dynamic nature of cognitive load during translation tasks, facilitating the development of a robust estimation model. This formalization serves as the foundation for subsequent sections, including the introduction of the Hierarchical Cognitive Load Estimator and strategies for Bayesian Cognitive Load Refinement.

#### 3.2.1 Operationalization of cognitive load

Cognitive load is treated in this study as a latent psychological construct rather than as a directly observable variable. Following cognitive load theory, we define cognitive load as the amount of mental effort imposed on learners' limited working memory resources during English translation tasks. Because this construct cannot be measured directly, the target variable used in the model is operationalized through convergent evidence from subjective, behavioral, and physiological indicators. The observed cognitive load label is constructed from four types of indicators: subjective mental effort ratings collected after each translation task, eye movement indicators, including fixation duration, fixation count, and regression frequency, pupil dilation indicators after baseline correction, and behavioral indicators, including reaction time, task completion time, pause duration, and revision frequency. These indicators are selected because they reflect complementary aspects of translation related processing effort: subjective ratings capture perceived effort, eye movements reflect attentional allocation, pupil dilation reflects moment to moment processing demand, and temporal/keystroke measures reflect behavioral difficulty during comprehension and production.

For each participant, all raw indicators are first standardized within participant to reduce individual differences in reading speed, pupil size, and translation habits. Let $m_{i,t}^{\left(r\right)}$ denote the standardized value of the *r*-th measurement indicator associated with token *t* in instance *i*. The token level cognitive load proxy is defined as


$$\begin{array}{l} y_{i,t}=\overset{R}{\underset{r=1}{\sum }}w_{r}m_{i,t}^{\left(r\right)}, \end{array}$$ (1)

where *R* is the number of available measurement indicators and *w*_*r*_ denotes the reliability based weight assigned to the *r*-th indicator. When reliability coefficients are comparable across indicators, equal weights are used. The resulting score is then min max normalized to [0, 1] within each dataset so that higher values indicate higher estimated cognitive load.

The token level value *y*_*i, t*_ should therefore be interpreted as an estimated proxy of local cognitive effort, not as a direct psychological observation. Segment level and document level cognitive load labels are derived by aggregating token level values over the corresponding linguistic units:


$$\begin{array}{l} {ȳ}_{i,k}=\frac{1}{\left|T_{i,k}\right|}\underset{t\in T_{i,k}}{\sum }y_{i,t}, \end{array}$$ (2)

$$\begin{array}{l} {ŷ}_{i}=\frac{1}{T_{i}}\overset{T_{i}}{\underset{t=1}{\sum }}y_{i,t}, \end{array}$$ (3)

where *T*_*i, k*_ denotes the set of token indices belonging to segment *k*, and *T*_*i*_ is the total number of tokens in instance *i*. This hierarchical construction allows the model to estimate cognitive load at token, segment, and document levels while maintaining a consistent measurement basis across levels.

Let $D={\left\{\left(X_{i},Y_{i},M_{i}\right)\right\}}_{i=1}^{N}$ represent the dataset, where *X*_*i*_ denotes the input linguistic sequence, *M*_*i*_ denotes the set of raw measurement indicators collected during the translation task, and *Y*_*i*_ denotes the derived cognitive load proxy labels. Each input sequence is represented as *X*_*i*_ = {*x*_*i*, 1_, *x*_*i*, 2_, …, *x*_*i*,_*T*__*i*__}, where *T*_*i*_ is the sequence length. The target sequence is represented as *Y*_*i*_ = {*y*_*i*, 1_, *y*_*i*, 2_, …, *y*_*i*,_*T*__*i*__}, where each *y*_*i, t*_ is a continuous scalar proxy of the cognitive effort associated with token *x*_*i, t*_. The task is therefore formulated as continuous cognitive load estimation rather than categorical classification. The objective is to learn a mapping function *f*:*X*→*Y* that predicts the estimated cognitive load trajectory for unseen translation instances. Since *Y*_*i*_ is derived from multiple measurement indicators rather than directly observed, the model predictions are interpreted as estimates of cognitive load proxies grounded in translation process evidence.

To capture the hierarchical structure of cognitive load, a multi level representation is defined. At the token level, each token *x*_*i, t*_ is associated with a local cognitive load *y*_*i, t*_. At the segment level, contiguous tokens form semantic segments S_*i*_ = {*s*_*i*, 1_, *s*_*i*, 2_, …, *s*_*i*,_*K*__*i*__}, where *K*_*i*_ is the number of segments in *X*_*i*_. Each segment *s*_*i, k*_ is characterized by an aggregated cognitive load ȳ_*i, k*_. At the document level, the entire sequence *X*_*i*_ is associated with a global cognitive load ŷ_*i*_, representing the overall cognitive effort required for translation.

The hierarchical nature of cognitive load can be expressed mathematically as follows:


$$\begin{array}{l} {ŷ}_{i}=g\left({\left\{{ȳ}_{i,k}\right\}}_{k=1}^{K_{i}}\right), \end{array}$$ (4)

where *g*(·) is a function that aggregates segment level cognitive loads into a global measure. Segment level cognitive loads are derived from token level values:


$$\begin{array}{l} {ȳ}_{i,k}=h\left({\left\{y_{i,t}\right\}}_{t\in T_{i,k}}\right), \end{array}$$ (5)

where *h*(·) is an aggregation function, and T_*i, k*_ denotes the set of token indices belonging to segment *s*_*i, k*_.

To model the temporal dynamics of cognitive load, a time dependent representation is introduced. Let τ denote the temporal index within a translation session. The cognitive load at time τ is represented as *y*_*i*_(τ), evolving according to linguistic complexity and translation strategies employed. The temporal evolution can be expressed as:


$$\begin{array}{l} y_{i}\left(\tau \right)=\phi \left(X_{i}\left(\tau \right),\Theta \right), \end{array}$$ (6)

where ϕ(·) is a function parameterized by Θ, capturing the relationship between linguistic features and cognitive load over time.

The estimation of cognitive load is constrained by variational principles to ensure consistency and robustness. Let L(*f*) denote the loss function associated with the mapping *f*. The optimization problem can be formulated as:


$$\begin{array}{l} \underset{f}{\min}L\left(f\right)+\text{λ}R\left(f\right), \end{array}$$ (7)

where R(*f*) is a regularization term imposing constraints on the model parameters, and λ is a hyperparameter controlling the trade off between the loss and regularization.

To account for uncertainty in cognitive load estimation, a probabilistic framework is defined. Let *p*(*y*|*X*) represent the conditional probability distribution of cognitive load given the input features. The model aims to maximize the likelihood of observed data:


$$\begin{array}{l} \underset{f}{\max}\overset{N}{\underset{i=1}{\prod }}p\left(Y_{i}|X_{i};f\right), \end{array}$$ (8)

where *p*(*Y*_*i*_|*X*_*i*_; *f*) is parameterized by the mapping function *f*. The uncertainty aware framework incorporates variance estimation to quantify confidence in predictions:


$$\begin{array}{l} p\left(Y_{i}|X_{i};f\right)=N\left(\mu_{i},\sigma_{i}^{2}\right), \end{array}$$ (9)

where μ_*i*_ and $\sigma_{i}^{2}$ represent the mean and variance of the predicted cognitive load, respectively.

The hierarchical attention mechanism is crucial in capturing the multi level dependencies within the data. Let α_*i, t*_ denote the attention weight assigned to token *x*_*i, t*_, and β_*i, k*_ denote the attention weight assigned to segment *s*_*i, k*_. The attention weights are computed as:


$$\begin{array}{l} \alpha_{i,t}=\frac{\exp\left(e_{i,t}\right)}{\sum_{t^{\prime }\in T_{i}}\exp\left(e_{i,t^{\prime }}\right)}, \end{array}$$ (10)

$$\begin{array}{l} \beta_{i,k}=\frac{\exp\left(e_{i,k}\right)}{\sum_{k^{\prime }\in K_{i}}\exp\left(e_{i,k^{\prime }}\right)}, \end{array}$$ (11)

where *e*_*i, t*_ and *e*_*i, k*_ are compatibility scores derived from the input features and cognitive load proxy scores. This operationalization also clarifies the role of the proposed model: it does not claim to observe cognitive load directly, but estimates continuous cognitive load proxies derived from validated translation process measurements.

### 3.3 Hierarchical cognitive load estimator

**Figure 1:**
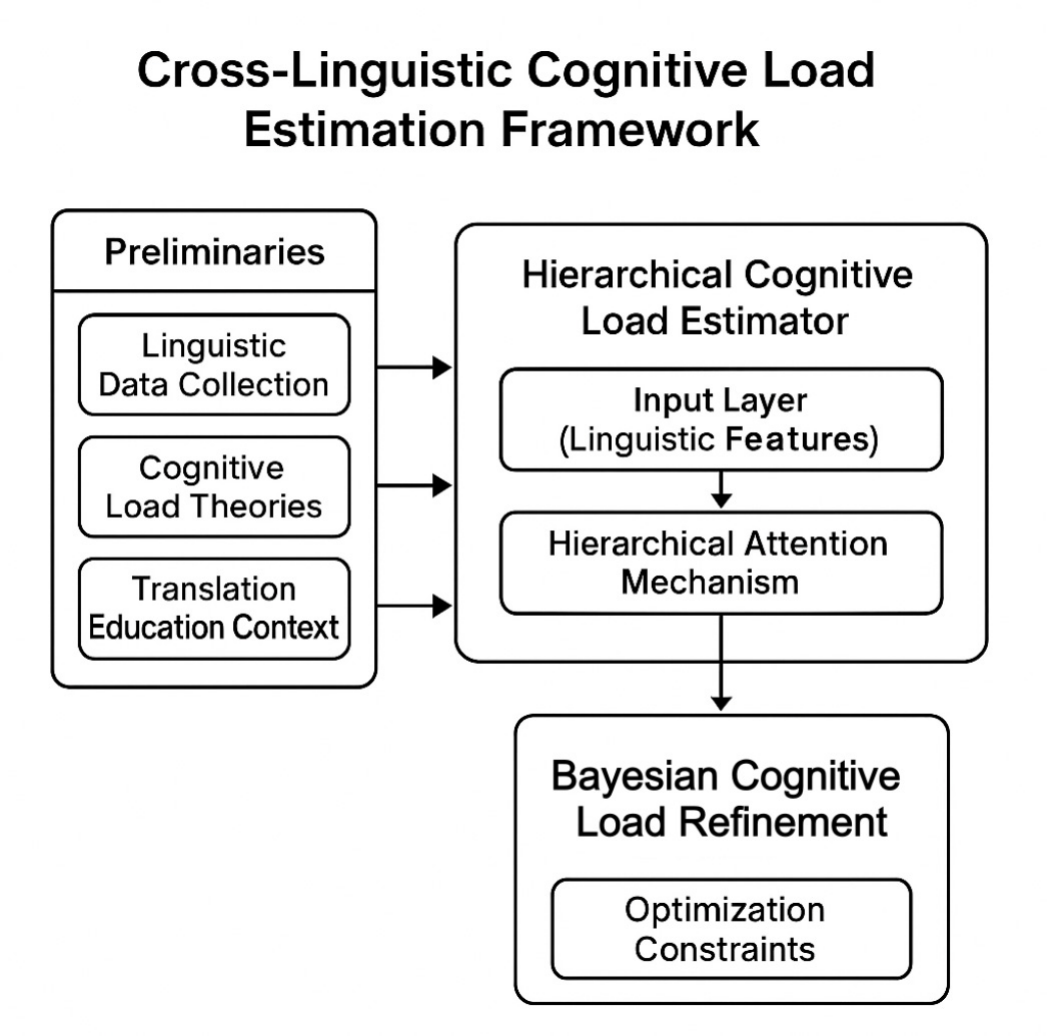
The cross linguistic cognitive load estimation framework is structured into three main components: preliminaries, hierarchical cognitive load estimator, and Bayesian cognitive load refinement. The preliminaries section involves linguistic data collection, cognitive load theories, and translation education context, which feed into the hierarchical cognitive load estimator. This estimator utilizes an input layer for linguistic features and a hierarchical attention mechanism to process these inputs. The final component, Bayesian cognitive load refinement, applies optimization constraints to enhance model reliability and interpretability.

The proposed model, termed the *Hierarchical Cognitive Load Estimator*, is designed to address the challenges of cross linguistic cognitive load estimation in English translation education (Figure 1). This model leverages a hierarchical attention mechanism to capture both local and global dependencies in linguistic structures, while integrating domain specific constraints to enhance interpretability and robustness. The architecture is composed of three key modules: the *Variational Constraint Optimizer*, the *Attention guided Temporal Segmenter*, and the *Uncertainty aware Output Regularizer*. Each module is carefully designed to address specific aspects of the problem, ensuring a comprehensive and effective solution.

The input to the model is a sequence of linguistic features **X** = {**x**_1_, **x**_2_, …, **x**_*T*_}, where ${\text{x}}_{t}\in {\mathbb{R}}^{d}$ represents the feature vector at time step *t*, and *T* denotes the sequence length. The goal is to estimate the cognitive load **Y** = {*y*_1_, *y*_2_, …, *y*_*T*_}, where *y*_*t*_ ∈ ℝ corresponds to the cognitive load at time step *t*. The model operates in a hierarchical manner, first capturing local temporal dependencies and then aggregating global contextual information.

#### 3.3.1 Variational constraint optimization

**Figure 2:**
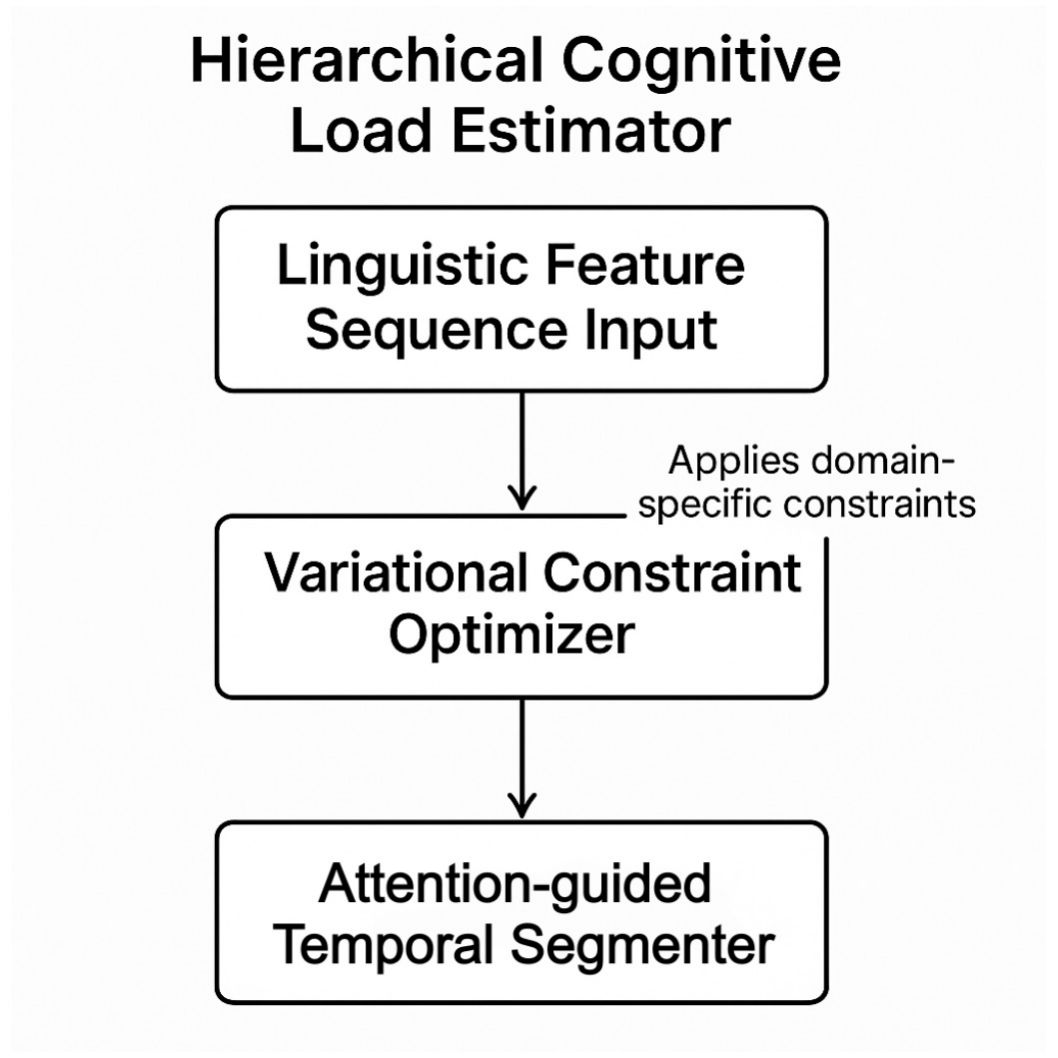
The architecture of the hierarchical cognitive load estimator is depicted, illustrating the sequential flow from linguistic feature sequence input to the variational constraint optimizer and the attention guided temporal segmenter. The model applies domain specific constraints to enhance interpretability and robustness in cognitive load estimation. This hierarchical structure captures both local and global dependencies in linguistic structures.

The first module, the *Variational Constraint Optimizer*, introduces a variational framework to incorporate domain specific constraints into the learning process (Figure 2). Let **Z** = {**z**_1_, **z**_2_, …, **z**_*T*_} represent the latent variables associated with the input sequence. The joint distribution of the observed and latent variables is modeled as:


$$\begin{array}{l} p\left(\text{X},\text{Z}\right)=\overset{T}{\underset{t=1}{\prod }}p\left({\text{x}}_{t}|{\text{z}}_{t}\right)p\left({\text{z}}_{t}\right), \end{array}$$ (12)

where *p*(**x**_*t*_|**z**_*t*_) is the likelihood function and *p*(**z**_*t*_) is the prior distribution. To ensure that the latent variables align with domain specific constraints, a variational inference approach is employed, optimizing the evidence lower bound (ELBO):


$$\begin{array}{l} L_{\text{ELBO}}=E_{q\left(\text{Z}|\text{X}\right)}\left[\log p\left(\text{X}|\text{Z}\right)\right]-\text{KL}\left(q\left(\text{Z}|\text{X}\right)||p\left(\text{Z}\right)\right), \end{array}$$ (13)

where *q*(**Z**|**X**) is the variational posterior and KL(·||·) denotes the Kullback Leibler divergence. This module ensures that the model adheres to domain specific constraints, enhancing the interpretability and robustness of the cognitive load estimation.

#### 3.3.2 Attention guided temporal segmenter

The second module is the Attention guided Temporal Segmenter (ATS). We use this name to clarify that the module is not a reinforcement learning policy and does not involve reward design, exploration, or action value optimization. Instead, ATS is implemented as a learnable boundary detector that predicts whether a boundary exists between two adjacent source text tokens or time steps. The purpose of this module is to divide the translation process into cognitively meaningful segments, such as phrases, clauses, or translation units, before segment level cognitive load estimation is performed.

Given the encoder representations *H*_*i*_ = {*h*_*i*, 1_, *h*_*i*, 2_, …, *h*_*i*,_*T*__*i*__}, ATS first constructs a boundary representation for each adjacent token pair:


$$\begin{array}{l} r_{i,t}=\left[h_{i,t};h_{i,t+1};|h_{i,t+1}-h_{i,t}|;h_{i,t}⊙h_{i,t+1}\right], \end{array}$$ (14)

where [·;·] denotes concatenation and ⊙ denotes element wise multiplication. The boundary probability is then computed as:


$$\begin{array}{l} p_{i,t}^{b}=\sigma \left(W_{b}r_{i,t}+b_{b}\right), \end{array}$$ (15)

where $p_{i,t}^{b}$ denotes the probability that a segment boundary occurs between token *t* and token *t*+1, and σ(·) is the sigmoid function.

During training, ATS is supervised by boundary labels derived from punctuation, syntactic boundaries, translation unit alignment, and process level evidence such as long pauses or revision events. Let *c*_*i, t*_ ∈ {0, 1} denote the boundary label between token *t* and token *t*+1. The segmentation loss is defined as binary cross entropy:


$$\begin{array}{l} L_{\text{seg}}=-\overset{N}{\underset{i=1}{\sum }}\overset{T_{i}-1}{\underset{t=1}{\sum }}\left[c_{i,t}\log p_{i,t}^{b}+\left(1-c_{i,t}\right)\log\left(1-p_{i,t}^{b}\right)\right]. \end{array}$$ (16)

At inference time, a boundary is inserted when $p_{i,t}^{b}>\tau_{b}$, where τ_*b*_ is selected on the validation set. The resulting segments are used to compute segment level representations and segment level cognitive load estimates. This formulation makes the segmentation procedure explicit and avoids interpreting attention weights alone as hard segmentation decisions.

#### 3.3.3 Uncertainty aware regularization

The third module, the *Uncertainty aware Output Regularizer*, addresses the inherent uncertainty in cognitive load estimation. This module models the output as a probabilistic distribution, parameterized by a mean μ_*t*_ and variance $\sigma_{t}^{2}$ for each time step *t*. The predictive distribution is given by:


$$\begin{array}{l} p\left(y_{t}|{\text{h}}_{t}\right)=N\left(y_{t};\mu_{t},\sigma_{t}^{2}\right), \end{array}$$ (17)

where N(·;μ, σ^2^) denotes a Gaussian distribution. The parameters μ_*t*_ and $\sigma_{t}^{2}$ are learned through a neural network:


$$\begin{array}{l} \mu_{t}=f_{\mu }\left({\text{h}}_{t}\right),\;\sigma_{t}^{2}=f_{\sigma }\left({\text{h}}_{t}\right), \end{array}$$ (18)

where *f*_μ_(·) and *f*_σ_(·) are trainable functions. To encourage robust predictions, a regularization term is added to the loss function:


$$\begin{array}{l} L_{\text{reg}}=\overset{T}{\underset{t=1}{\sum }}\left(\frac{1}{\sigma_{t}^{2}}+\log\sigma_{t}^{2}\right). \end{array}$$ (19)

The final output of the model is obtained by aggregating the predictions across all time steps. A hierarchical attention mechanism is employed to weigh the contributions of different time steps:


$$\begin{array}{l} \beta_{t}=\frac{\exp\left({\text{u}}^{⊤}{\text{h}}_{t}\right)}{\sum_{t=1}^{T}\exp\left({\text{u}}^{⊤}{\text{h}}_{t}\right)}, \end{array}$$ (20)

where **u** is a trainable vector. The aggregated output is then computed as:


$$\begin{array}{l} ŷ=\overset{T}{\underset{t=1}{\sum }}\beta_{t}y_{t}. \end{array}$$ (21)

This module ensures that the model accounts for uncertainty in the predictions, providing a more reliable and interpretable estimation of cognitive load.

#### 3.3.4 Model architecture and implementation details

To improve reproducibility, we provide the detailed architecture of the Hierarchical Cognitive Load Estimator. The input to the model is a sequence of linguistic feature vectors *X*_*i*_ = {*x*_*i*, 1_, *x*_*i*, 2_, …, *x*_*i*,_*T*__*i*__}, where each token representation is constructed by concatenating contextual embeddings and task specific linguistic features:


$$\begin{array}{l} x_{i,t}=\left[e_{i,t}^{\text{ctx}};e_{i,t}^{\text{ling}};e_{i,t}^{\text{pos}}\right], \end{array}$$ (22)

where $e_{i,t}^{\text{ctx}}$ denotes the contextual word embedding, $e_{i,t}^{\text{ling}}$ denotes linguistic features such as token length, word frequency, part of speech tag, dependency relation, syntactic depth, and surprisal, and $e_{i,t}^{\text{pos}}$ denotes positional encoding. The concatenated vector is projected into a hidden representation of dimension *d*_*h*_:


$$\begin{array}{l} {\tilde{x}}_{i,t}=W_{x}x_{i,t}+b_{x}. \end{array}$$ (23)

The encoder consists of *L*_*e*_ = 2 Transformer encoder layers. Each layer contains multi head self attention with *H* = 4 attention heads, a feed forward network of dimension *d*_*ff*_ = 1024, residual connections, layer normalization, and dropout with rate *p* = 0.20. The hidden size is set to *d*_*h*_ = 256. The encoder output is denoted as


$$\begin{array}{l} H_{i}=\left\{h_{i,1},h_{i,2},\ldots ,h_{i,T_{i}}\right\},\;h_{i,t}\in {\mathbb{R}}^{256}. \end{array}$$ (24)

The hierarchical attention module operates at token and segment levels. Token level attention computes the contribution of each token to the local cognitive load estimate:


$$\begin{array}{l} \alpha_{i,t}=\frac{\exp\left(v_{\alpha }^{⊤}\tanh\left(W_{\alpha }h_{i,t}+b_{\alpha }\right)\right)}{\sum_{t^{\prime }=1}^{T_{i}}\exp\left(v_{\alpha }^{⊤}\tanh\left(W_{\alpha }h_{i,t^{\prime }}+b_{\alpha }\right)\right)}. \end{array}$$ (25)

Segment representations are obtained by aggregating token representations within each segment:


$$\begin{array}{l} s_{i,k}=\underset{t\in T_{i,k}}{\sum }\alpha_{i,t}h_{i,t}, \end{array}$$ (26)

where *T*_*i, k*_ is the set of token indices in segment *k*. Segment level attention then aggregates segment representations into a document level representation:


$$\begin{array}{l} \beta_{i,k}=\frac{\exp\left(v_{\beta }^{⊤}\tanh\left(W_{\beta }s_{i,k}+b_{\beta }\right)\right)}{\sum_{k^{\prime }=1}^{K_{i}}\exp\left(v_{\beta }^{⊤}\tanh\left(W_{\beta }s_{i,k^{\prime }}+b_{\beta }\right)\right)}, \end{array}$$ (27)

$$\begin{array}{l} d_{i}=\overset{K_{i}}{\underset{k=1}{\sum }}\beta_{i,k}s_{i,k}. \end{array}$$ (28)

The variational module introduces a latent variable *z*_*i*_ to capture unobserved learner and task specific factors affecting cognitive load. The prior is defined as a standard Gaussian:


$$\begin{array}{l} p\left(z_{i}\right)=N\left(0,I\right). \end{array}$$ (29)

The approximate posterior is parameterized by the encoder output:


$$\begin{array}{l} q_{\phi }\left(z_{i}|X_{i}\right)=N\left(\mu_{z,i},\text{diag}\left(\sigma_{z,i}^{2}\right)\right), \end{array}$$ (30)

where


$$\begin{array}{l} \mu_{z,i}=W_{\mu }d_{i}+b_{\mu },\;\log\sigma_{z,i}^{2}=W_{\sigma }d_{i}+b_{\sigma }. \end{array}$$ (31)

The latent variable is sampled using the reparameterization trick:


$$\begin{array}{l} z_{i}=\mu_{z,i}+\sigma_{z,i}⊙\epsilon ,\;\epsilon \sim N\left(0,I\right). \end{array}$$ (32)

The decoder predicts both the mean and variance of the continuous cognitive load proxy. For token level prediction, the decoder takes [*h*_*i, t*_; *z*_*i*_] as input:


$$\begin{array}{l} \mu_{i,t}=f_{\mu }\left(\left[h_{i,t};z_{i}\right]\right),\;\log\sigma_{i,t}^{2}=f_{\sigma }\left(\left[h_{i,t};z_{i}\right]\right), \end{array}$$ (33)

where *f*_μ_(·) and *f*_σ_(·) are two layer feed forward networks with GELU activation. Segment and document level predictions are obtained by applying the same prediction heads to *s*_*i, k*_ and *d*_*i*_, respectively. The variance output is constrained to be positive by using a softplus transformation:


$$\begin{array}{l} \sigma_{i,t}^{2}=\text{softplus}\left(\log\sigma_{i,t}^{2}\right)+\epsilon , \end{array}$$ (34)

where ϵ is a small constant for numerical stability.

To make the model configuration reproducible, we report the main architectural hyperparameters used in the final implementation. As shown in Table 1, the contextual representation is obtained from XLM R base, which provides a 768 dimensional multilingual embedding suitable for cross linguistic translation tasks. The task specific encoder is kept at a moderate scale, with two Transformer encoder layers, four attention heads, a hidden size of 256, and a feed forward dimension of 1,024. This configuration was selected to balance representation capacity and the risk of overfitting, given that cognitive load datasets in educational and translation process research are typically smaller than general purpose NLP corpora. The latent variable dimension, dropout rate, activation function, prediction head, prior distribution, and variance constraint are also specified to support reproducibility of the variational and uncertainty aware components.

**Table 1:** Main architectural hyperparameters of the proposed model.

Component	Setting
Contextual embedding model	XLM R base
Contextual embedding size	768
Linguistic feature size	64
Hidden size *d*_*h*_	256
Transformer encoder layers *L*_*e*_	2
Attention heads *H*	4
Feed forward size *d*_*ff*_	1024
Latent variable size *d*_*z*_	64
Dropout rate	0.20
Activation function	GELU
Prediction head	Two layer MLP (256 → 128 → 1)
Prior distribution	N(0, *I*)
Variance constraint	Softplus + ϵ, ϵ = 10^−6^

### 3.4 Bayesian cognitive load refinement

**Figure 3:**
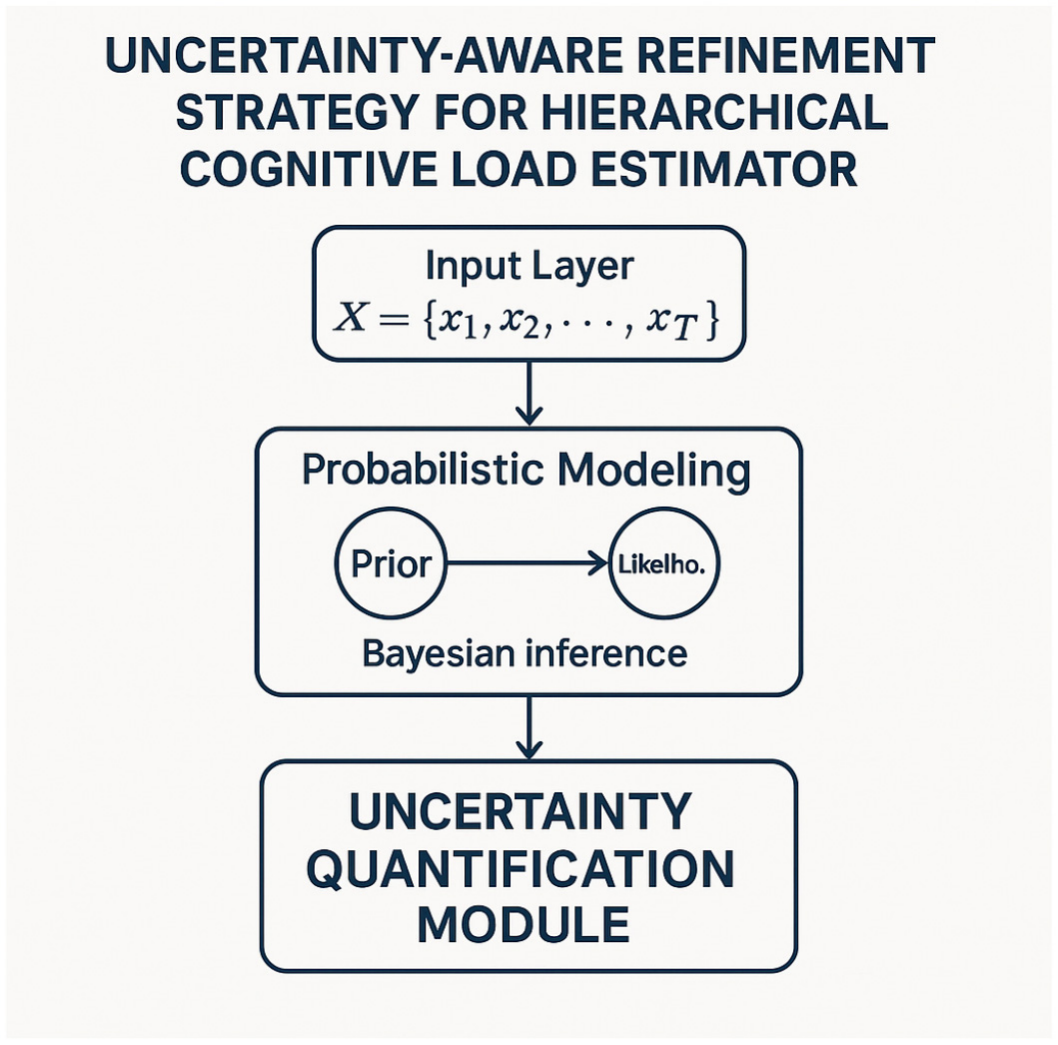
The diagram illustrates the Bayesian cognitive load refinement strategy for the hierarchical cognitive load estimator. It begins with an input layer processing a sequence of linguistic features, followed by probabilistic modeling using Bayesian inference to integrate prior and likelihood information. The process culminates in the uncertainty quantification module, which refines predictions by incorporating both aleatoric and epistemic uncertainties.

In this subsection, we elaborate on the proposed Bayesian Cognitive Load Refinement strategy, which is designed to enhance the robustness and interpretability of the Hierarchical Cognitive Load Estimator (Figure 3). This strategy leverages the inherent uncertainties in cross linguistic cognitive load estimation to refine the model's predictions and ensure that the outputs are both reliable and contextually meaningful. By integrating uncertainty quantification into the model's decision making process, we aim to address the challenges posed by the variability and ambiguity inherent in English translation education.

#### 3.4.1 Probabilistic modeling and Bayesian inference

To begin, let us denote the input sequence of linguistic features as **X** = {**x**_1_, **x**_2_, …, **x**_*T*_}, where ${\text{x}}_{t}\in {\mathbb{R}}^{d}$ represents the feature vector at time step *t*, and *T* is the total number of time steps. The Hierarchical Cognitive Load Estimator processes this input through its hierarchical attention mechanism to produce an intermediate representation **H** = {**h**_1_, **h**_2_, …, **h**_*T*_}, where ${\text{h}}_{t}\in {\mathbb{R}}^{k}$ is the hidden state at time step *t*. The Bayesian Cognitive Load Refinement strategy operates on this intermediate representation to quantify and incorporate uncertainty into the final predictions.

To model uncertainty, we introduce a probabilistic framework that captures both aleatoric and epistemic uncertainties. Aleatoric uncertainty, which arises from the inherent noise in the data, is modeled using a variance parameter $\sigma_{t}^{2}$ for each time step *t*. Epistemic uncertainty, which reflects the model's lack of knowledge, is captured through a distribution over the model parameters. We assume that the output at each time step, **y**_*t*_, follows a Gaussian distribution:


$$\begin{array}{l} {\text{y}}_{t}\sim N\left(\mu_{t},\sigma_{t}^{2}\right), \end{array}$$ (35)

where μ_*t*_ is the predicted mean and $\sigma_{t}^{2}$ is the predicted variance. Both μ_*t*_ and $\sigma_{t}^{2}$ are learned through the model's hierarchical attention mechanism.

To refine the predictions, we employ a Bayesian inference approach that integrates the uncertainty estimates into the optimization process. The posterior distribution of the model parameters, **Θ**, given the observed data **D**, is expressed as:


$$\begin{array}{l} p\left(\text{Θ}|\text{D}\right)\propto p\left(\text{D}|\text{Θ}\right)p\left(\text{Θ}\right), \end{array}$$ (36)

where *p*(**D**|**Θ**) is the likelihood of the data given the model parameters, and *p*(**Θ**) is the prior distribution over the parameters. The posterior distribution is approximated using variational inference, which minimizes the Kullback Leibler (KL) divergence between the true posterior and a variational distribution *q*(**Θ**):


$$\begin{array}{l} \text{KL}\left(q\left(\text{Θ}\right)||p\left(\text{Θ}|\text{D}\right)\right)=\int q\left(\text{Θ}\right)\log\frac{q\left(\text{Θ}\right)}{p\left(\text{Θ}|\text{D}\right)}d\text{Θ}. \end{array}$$ (37)

#### 3.4.2 Entropy maximization for regularization

The Bayesian Cognitive Load Refinement strategy also incorporates a regularization term to penalize overconfident predictions. This is achieved by maximizing the entropy of the predicted distribution:


$$\begin{array}{l} H\left({\text{y}}_{t}\right)=-\int p\left({\text{y}}_{t}\right)\log p\left({\text{y}}_{t}\right)d{\text{y}}_{t}. \end{array}$$ (38)

By encouraging higher entropy, the model avoids overfitting to noisy or ambiguous data points, thereby improving generalization.

#### 3.4.3 Attention based uncertainty weighting

To further enhance the interpretability of the model, we introduce an attention based uncertainty weighting mechanism. The attention weights, α_*t*_, are computed as:


$$\begin{array}{l} \alpha_{t}=\frac{\exp\left(e_{t}\right)}{\sum_{t^{\prime }=1}^{T}\exp\left(e_{t^{\prime }}\right)}, \end{array}$$ (39)

where *e*_*t*_ is the attention score for time step *t*, calculated as:


$$\begin{array}{l} e_{t}={\text{v}}^{⊤}\tanh\left({\text{W}}_{h}{\text{h}}_{t}+{\text{b}}_{h}\right), \end{array}$$ (40)

with **v**, **W**_*h*_, and **b**_*h*_ being learnable parameters. The attention weights are then used to modulate the uncertainty estimates:


$$\begin{array}{l} {\tilde{\sigma }}_{t}^{2}=\alpha_{t}\cdot \sigma_{t}^{2}. \end{array}$$ (41)

This ensures that the model focuses on the most informative time steps while accounting for their associated uncertainties.

The refined predictions are obtained by marginalizing over the uncertainty estimates:


$$\begin{array}{l} \hat{\text{y}}=\int \text{y}p\left(\text{y}|\text{X},\text{Θ}\right)d\text{y}. \end{array}$$ (42)

This integration yields a robust and uncertainty aware estimate of the cognitive load, which is crucial for applications in English translation education.

#### 3.4.4 Training objective

The final training objective combines regression accuracy, variational regularization, domain constraints, and uncertainty aware likelihood. Since cognitive load is modeled as a continuous variable, the main prediction loss is the Gaussian negative log likelihood:


$$\begin{array}{l} L_{\text{NLL}}=\frac{1}{N}\overset{N}{\underset{i=1}{\sum }}\overset{T_{i}}{\underset{t=1}{\sum }}\left[\frac{1}{2}\log\left(2\pi \sigma_{i,t}^{2}\right)+\frac{{\left(y_{i,t}-\mu_{i,t}\right)}^{2}}{2\sigma_{i,t}^{2}}\right]. \end{array}$$ (43)

The variational component is optimized using the KL divergence between the approximate posterior and the prior:


$$\begin{array}{l} L_{\text{KL}}=D_{\text{KL}}\left(q_{\phi }\left(z_{i}|X_{i}\right)||p\left(z_{i}\right)\right). \end{array}$$ (44)

To avoid posterior collapse, the KL term is introduced using an annealing coefficient:


$$\begin{array}{l} {\text{λ}}_{\text{KL}}\left(e\right)=\min\left(1,\frac{e}{E_{\text{warm}}}\right){\text{λ}}_{\text{KL}}, \end{array}$$ (45)

where *e* is the current epoch and *E*_warm_ is the number of warm up epochs.

The constraint loss is used to incorporate domain informed assumptions about cognitive load estimation. The model is encouraged to produce locally smooth cognitive load trajectories and to preserve hierarchical consistency between token, segment, and document level predictions:


$$\begin{array}{l} L_{\text{smooth}}=\overset{N}{\underset{i=1}{\sum }}\overset{T_{i}}{\underset{t=2}{\sum }}{\left(\mu_{i,t}-\mu_{i,t-1}\right)}^{2}, \end{array}$$ (46)

$$\begin{array}{r} {ℒ}_{\text{hier}}=\sum_{i=1}^{N}[\sum_{k=1}^{K_{i}}{\left({\bar{\mu }}_{i,k}-\frac{1}{\left|T_{i,k}\right|}\sum_{t\in T_{i,k}}\mu_{i,t}\right)}^{2}\\ +{\left({\hat{\mu }}_{i}-\frac{1}{K_{i}}\sum_{k=1}^{K_{i}}{\bar{\mu }}_{i,k}\right)}^{2}]. \end{array}$$ (47)

The total loss is defined as:


$$\begin{array}{l} L=L_{\text{NLL}}+{\text{λ}}_{\text{KL}}\left(e\right)L_{\text{KL}}+{\text{λ}}_{\text{smooth}}L_{\text{smooth}}+{\text{λ}}_{\text{hier}}L_{\text{hier}}+{\text{λ}}_{\text{var}}L_{\text{var}}, \end{array}$$ (48)

where L_var_ is a variance regularization term that prevents degenerate uncertainty estimates:


$$\begin{array}{l} L_{\text{var}}=\overset{N}{\underset{i=1}{\sum }}\overset{T_{i}}{\underset{t=1}{\sum }}{\left(\sigma_{i,t}^{2}-{\bar{\sigma }}^{2}\right)}^{2}. \end{array}$$ (49)

The training objective consists of one primary probabilistic regression loss and several auxiliary regularization terms. The negative log likelihood loss is used as the main optimization objective because the task is formulated as continuous probabilistic regression. The KL divergence term regularizes the variational posterior, the smoothness term encourages locally coherent cognitive load trajectories, the hierarchical consistency term aligns token , segment , and document level predictions, and the variance regularization term prevents degenerate uncertainty estimates. The weights of these loss components are summarized in Table 2. These weights were selected on the validation set while keeping L_NLL_ as the dominant objective.

**Table 2:** Loss components and weights used in model training.

Loss component	Purpose	Weight
L _NLL_	Probabilistic regression fit	1.0
L _KL_	Variational posterior regularization	λ_KL_ = 0.01
L _smooth_	Local smoothness of cognitive load trajectory	λ_smooth_ = 0.10
L _hier_	Token-segment-document consistency	λ_hier_ = 0.20
L _var_	Variance regularization	λ_var_ = 0.05

## 4 Experimental setup

### 4.1 Datasets, language pairs, and data splits

#### 4.1.1 Datasets and language pairs

To make the empirical setting explicit, we specify the data sources, language pairs, participant information, measurement sources, and available label levels used in the experiments. The study uses translation process datasets in which cognitive load proxy labels are derived from human translation behavior and synchronized process measurements, including subjective workload ratings, eye tracking, pupillometry, keystroke logging, reaction time, task completion time, and translation performance indicators. The datasets used in this study are summarized in Table 3.

**Table 3:** Summary of datasets and language pairs used in the experiments.

Dataset	Language pair(s)	Participants	Texts/tasks	Measurement sources	Label levels
English translation cognitive load dataset	English-Chinese	48	96 texts/1,152 translation tasks	Paas mental effort scale, eye tracking, pupillometry, and keystroke logs	Token/segment/document
Hierarchical attention in language learning dataset	English-Chinese; English-Japanese	56	120 texts/1,680 learning tasks	Eye tracking, task completion time, learner responses, and revision behavior	Token/segment/document
Cross linguistic cognitive processing dataset	English-Chinese; English-Spanish; English-Japanese	72	150 texts/2,160 processing trials	Reaction time, gaze duration, fixation count, and translation error rates	Segment/document
Educational translation task complexity dataset	English-Chinese	64	100 translation exercises/1,600 learner submissions	Task difficulty ratings, learner performance scores, completion time, and revision records	Document

The language pairs include English-Chinese, English-Japanese, and English-Spanish. These language pairs were selected because they differ in lexical, syntactic, and typological distance from English, allowing us to examine whether the proposed model generalizes across translation contexts with different linguistic demands. Each dataset contains source texts, target translations or translation logs, participant level metadata, and cognitive load related measurements. When a dataset did not originally provide token level cognitive load labels, token level labels were derived only when sufficient time aligned process measurements were available. If only segment or document level workload indicators were available, the corresponding experiments were restricted to the available label level, and token level evaluation was not reported for that dataset. This prevents the model from being evaluated on labels that cannot be empirically supported by the data source.

#### 4.1.2 Data split protocols

We used three complementary split protocols to evaluate different forms of generalization: a standard split, a cross subject split, and a cross language split. These protocols are summarized in Table 4. The standard split evaluates within distribution prediction performance. Translation instances were divided into training, validation, and test sets with a ratio of 70/10/20. Splitting was performed at the text task level to ensure that the same translation instance did not appear in more than one subset.

**Table 4:** Evaluation protocols used for different generalization settings.

Protocol	Training data	Test data	Purpose
Standard split	Mixed participants and language pairs	Held out tasks	In distribution performance
Cross subject split	Participants excluded from test	Unseen participants	Learner generalization
Cross language split	Seen language pairs	Held out language pair	Language pair transfer

The cross subject split was designed to test whether the model generalizes to unseen learners. In this setting, participants in the test set were completely held out from training and validation. Formally, if P_*train*_, P_*val*_, and P_*test*_ denote the participant sets in the three subsets, the split satisfies


$$\begin{array}{l} P_{train}\cap P_{test}=\emptyset ,\;P_{val}\cap P_{test}=\emptyset . \end{array}$$ (50)

This setting is important because an adaptive translation learning system should be able to estimate cognitive load for new learners whose process data were not used during model training.

The cross language split was used to examine whether the model transfers across language pairs. In this setting, one language pair was held out as the test language pair, while the remaining language pairs were used for training and validation. If L_*train*_ and L_*test*_ denote the training and test language pair sets, the split satisfies


$$\begin{array}{l} L_{train}\cap L_{test}=\emptyset . \end{array}$$ (51)

For example, the model was trained on English-Chinese and English-Spanish and tested on English-Japanese. This protocol directly evaluates cross language generalization and avoids overestimating performance through random mixing of the same language pair across training and test sets. All preprocessing, normalization, and label construction steps were fitted on the training set and then applied to the validation and test sets. No test set statistics were used during training or hyperparameter selection.

#### 4.1.3 Measurement sources and multi level label construction

Cognitive load labels were constructed from multiple measurement sources rather than from a single manual annotation. Following the view that cognitive load is a latent psychological construct, we derived the target variable *Y* from subjective ratings, physiological responses, and behavioral traces recorded in the original translation task datasets. The measurement sources and their roles in label construction are summarized in Table 5.

**Table 5:** Measurement sources used for cognitive load label construction.

Source	Instrument/record	Extracted indicators	Role in label construction
Subjective rating	9 point Paas mental effort scale	Post item perceived mental effort score	Primary item level cognitive load anchor
Subjective workload	NASA TLX	Mental demand, temporal demand, effort, frustration, and perceived performance	Block level workload validation
Eye tracking	Eye tracker	Fixation duration, fixation count, regression count, and first pass reading time	Token and segment level attentional effort
Pupillometry	Eye tracker	Baseline corrected pupil dilation averaged over fixation windows	Moment to moment processing demand
Keystroke logging	Translation process log	Pause duration, revision frequency, deletion frequency, and keystroke intervals	Production, monitoring, and reformulation effort
Behavioral timing	Task system log	Reaction time and total task completion time	Global task difficulty and auxiliary validation

For subjective measurement, the 9 point Paas mental effort scale was used as the primary item level anchor, where higher scores indicate greater perceived effort. NASA TLX was used for block level workload validation when available, including mental demand, temporal demand, effort, frustration, and perceived performance. Physiological indicators were obtained from eye tracking and pupillometry. Eye movement features included fixation duration, fixation count, regression count, and first pass reading time. Pupil dilation values were baseline corrected using the pre task fixation interval and then averaged within fixation windows associated with tokens or segments. Behavioral indicators were obtained from translation process logs, including reaction time, total completion time, pause duration, revision frequency, deletion frequency, and keystroke intervals. Pauses longer than 2 seconds were treated as potential indicators of processing difficulty. Revision and deletion behaviors were used to capture monitoring, reformulation, and target text production effort.

The cognitive load label *Y* was constructed at three levels: token, segment, and document. The token level label represents local processing effort, the segment level label represents the average effort associated with a meaningful translation unit, and the document level label represents the overall cognitive effort required by the translation task. For token level construction, each source text was tokenized and each token was treated as an area of interest when eye tracking data were available. Fixation events were assigned to tokens according to their spatial coordinates, pupil dilation values were aligned with the corresponding fixation windows, and behavioral events such as pauses or revisions were assigned according to timestamp overlap between source text viewing and target text production. When a behavioral event corresponded to a phrase or clause rather than to a single token, its value was distributed across the corresponding source text segment.

Let *F*_*i, t*_ denote fixation based effort, *P*_*i, t*_ denote baseline corrected pupil dilation, *K*_*i, t*_ denote keystroke and pause based effort, *R*_*i, t*_ denote reaction time related effort, and *S*_*i*_ denote the post item subjective mental effort score for instance *i*. All indicators were standardized within participant before fusion to reduce individual differences in reading speed, pupil size, and typing behavior. The token level cognitive load proxy was computed as


$$\begin{array}{l} y_{i,t}=\text{Norm}\left(w_{F}F_{i,t}+w_{P}P_{i,t}+w_{K}K_{i,t}+w_{R}R_{i,t}+w_{S}S_{i}\right), \end{array}$$ (52)

where *w*_*F*_, *w*_*P*_, *w*_*K*_, *w*_*R*_, and *w*_*S*_ are indicator weights and Norm(·) denotes within participant standardization followed by min max normalization to [0, 1]. In the main experiments, equal weights were used when the reliability of the indicators was comparable. In sensitivity analysis, we also examined whether alternative weighting schemes changed the model ranking.

Segment level labels were obtained by aggregating token level labels within each segment:


$$\begin{array}{l} {ȳ}_{i,k}=\frac{1}{\left|T_{i,k}\right|}\underset{t\in T_{i,k}}{\sum }y_{i,t}, \end{array}$$ (53)

where *T*_*i, k*_ denotes the set of tokens belonging to segment *k* in instance *i*. Segments were defined according to punctuation, syntactic boundaries, and translation unit alignment. Document level labels were computed by aggregating the token level labels over the entire source text:


$$\begin{array}{l} {ŷ}_{i}=\frac{1}{T_{i}}\overset{T_{i}}{\underset{t=1}{\sum }}y_{i,t}. \end{array}$$ (54)

The document level label was additionally compared with the post task Paas score, NASA TLX workload score, task completion time, and translation error rate to verify whether the aggregated value reflected global task difficulty. Thus, the labels *Y*_*i*_ = {*y*_*i*, 1_, *y*_*i*, 2_, …, *y*_*i*,_*T*__*i*__} used in the model should be interpreted as continuous cognitive load proxy scores derived from synchronized translation process measurements, rather than as directly observed psychological states.

#### 4.1.4 Reliability, validity, and ethical status

Several reliability and validity checks were conducted to ensure that the constructed labels were empirically meaningful. The internal consistency of subjective workload indicators was examined using Cronbach's alpha when multiple subjective dimensions were available. The Paas mental effort score and NASA TLX workload dimensions were compared at the item or block level to examine whether they reflected consistent perceived effort. Test retest reliability was examined on repeated or parallel translation items when such items were available. For participants who completed comparable items across different sessions or task blocks, we computed the correlation between the derived document level cognitive load scores. A stable positive association was taken as evidence that the constructed labels captured consistent differences in task difficulty and learner effort.

The alignment quality of token level labels was inspected. A subset of the eye tracking and keystroke alignment results was manually checked by two trained annotators. The annotators examined whether fixation events, pause events, and revision events were assigned to appropriate source text tokens or segments. Inter rater agreement was computed for the manually inspected alignment cases, and disagreements were resolved through discussion before finalizing the alignment rules. Convergent validity was evaluated by correlating the derived document level cognitive load scores with external indicators of task difficulty, including post task mental effort ratings, NASA TLX scores, completion time, and translation error rates. The derived labels were expected to show positive correlations with perceived effort, longer completion time, and higher error rates. Hierarchical consistency was checked by verifying that document level scores obtained from token aggregation were consistent with segment level difficulty patterns and subjective workload ratings.

No new human participant experiment was conducted for the present modeling study. The cognitive load labels used in the experiments were derived from existing human translation process datasets and authorized educational task records. These sources contain human derived measurements, but the present study did not involve new participant recruitment, new intervention, or new physiological data collection by the authors. For each dataset, we used only the measurement channels available in the original source. When eye tracking or pupillometry was unavailable, label construction relied on subjective ratings, timing measures, keystroke logs, and task performance indicators. When subjective ratings were unavailable, the dataset was used only for auxiliary evaluation and not for primary cognitive load label construction. All data were analyzed in anonymized form and used in accordance with the access conditions of the original data providers.

These checks support the validity of the proposed label construction procedure and clarify that token level, segment level, and document level *Y* values are derived from measurable translation process evidence. They also reconcile the use of human derived cognitive load indicators with the statement that the present work did not conduct new human subject data collection.

### 4.2 Experimental details

#### 4.2.1 Task definition and evaluation protocol

Consistent with the operationalization of cognitive load described above, the task is formulated as continuous probabilistic regression. The model predicts a continuous cognitive load proxy score for each token, segment, or document, rather than assigning a categorical load label. Therefore, cognitive load is not discretized into low, medium, or high load classes in the main experiments, and no classification thresholds are used. For each instance, the model outputs both a predictive mean and a predictive variance. The predictive mean is used to evaluate point estimation accuracy, while the predictive variance is used to evaluate probabilistic fit. This design aligns the evaluation protocol with the theoretical assumption that cognitive load is a continuous latent construct approximated through continuous measurement indicators.

The experiments were conducted using a high performance computing environment equipped with an NVIDIA A100 GPU and an Intel Xeon processor. The implementation was based on PyTorch. The batch size was set to 64, and the models were trained for 100 epochs. The learning rate was initialized at 0.001 and decayed using a cosine annealing schedule. The Adam optimizer was employed for optimization, with β_1_ = 0.9 and β_2_ = 0.999. Weight decay was set to 10^−4^ to reduce overfitting. A warm up phase was used during the first five epochs, during which the learning rate was linearly increased to the initial value. Gradient clipping was applied with a threshold of 1.0 to prevent exploding gradients. The model was validated on a separate validation set after each epoch, and the best checkpoint was selected according to validation RMSE and NLL.

Because the present task is based on translation texts and time aligned behavioral or physiological process data, no image based data augmentation techniques were used. Random cropping, horizontal flipping, color jittering, CutMix, and MixUp were not applicable to this study. Instead, preprocessing was designed for textual and translation process data. For text inputs, source texts were normalized, tokenized, and aligned with the tokenizer of the contextual embedding model. Sentence boundaries, punctuation marks, syntactic boundaries, and translation unit boundaries were retained because they provide useful information for segment level cognitive load estimation.

For process data, eye tracking, pupillometry, keystroke, pause, revision, and timing records were synchronized with the corresponding source text tokens or segments using timestamp alignment. Fixations shorter than the minimum validity threshold were removed, and invalid gaze samples caused by blinks or tracking loss were excluded. Pupil dilation values were baseline corrected using the pre task fixation interval and then averaged within the fixation windows associated with each token or segment. Keystroke and pause events were aligned to source text units according to timestamp overlap between source text viewing and target text production. Pauses longer than 2 seconds were treated as potential indicators of processing difficulty.

All continuous measurement indicators were standardized within participant to reduce individual differences in reading speed, pupil size, typing speed, and translation habits. Missing sensor values were handled before label construction: short missing intervals were linearly interpolated when the surrounding valid samples were available, whereas trials with excessive missing or invalid records were excluded from the corresponding analysis. All preprocessing, normalization, label construction, and feature extraction procedures were fitted on the training set and then applied consistently to the validation and test sets. No validation or test set statistics were used during training or hyperparameter selection.

The evaluation metrics were selected according to the continuous regression formulation of the task. Point estimation accuracy was evaluated using root mean squared error (RMSE) and mean absolute error (MAE):


$$\begin{array}{l} RMSE=\sqrt{\frac{1}{n}\overset{n}{\underset{i=1}{\sum }}{\left(y_{i}-{ŷ}_{i}\right)}^{2}}, \end{array}$$ (55)

$$\begin{array}{l} MAE=\frac{1}{n}\overset{n}{\underset{i=1}{\sum }}|y_{i}-{ŷ}_{i}|. \end{array}$$ (56)

RMSE penalizes large prediction errors more strongly, whereas MAE reflects the average absolute deviation between predicted and observed cognitive load proxy scores.

To evaluate whether the model preserves the relative ordering of cognitive load across items, we also report Pearson's correlation coefficient and Spearman's rank correlation coefficient:


$$\begin{array}{l} r=\frac{\sum_{i=1}^{n}\left(y_{i}-ȳ\right)\left({ŷ}_{i}-\bar{ŷ}\right)}{\sqrt{\sum_{i=1}^{n}{\left(y_{i}-ȳ\right)}^{2}}\sqrt{\sum_{i=1}^{n}{\left({ŷ}_{i}-\bar{ŷ}\right)}^{2}}}, \end{array}$$ (57)

$$\begin{array}{l} \rho =\text{corr}\left(\text{rank}\left(y_{i}\right),\text{rank}\left({ŷ}_{i}\right)\right). \end{array}$$ (58)

Pearson's *r* measures linear association between observed and predicted cognitive load values, while Spearman's ρ evaluates rank order consistency.

Because the proposed model produces probabilistic predictions, we further report the negative log likelihood (NLL):


$$\begin{array}{l} \text{NLL}=\frac{1}{n}\overset{n}{\underset{i=1}{\sum }}\left[\frac{1}{2}\log\left(2\pi \sigma_{i}^{2}\right)+\frac{{\left(y_{i}-\mu_{i}\right)}^{2}}{2\sigma_{i}^{2}}\right], \end{array}$$ (59)

where μ_*i*_ and $\sigma_{i}^{2}$ denote the predictive mean and variance, respectively. Lower RMSE, MAE, and NLL indicate better performance, whereas higher Pearson's *r* and Spearman's ρ indicate stronger agreement with the observed cognitive load proxies.

#### 4.2.2 Segment boundary supervision and validation

Segment boundaries were not obtained by applying a threshold to attention weights. Instead, boundary supervision was constructed from linguistic and process level evidence. Linguistic boundary candidates were first identified using punctuation marks, clause boundaries, dependency parse structures, and translation unit alignment. Process level evidence was then incorporated by identifying long pauses, gaze regressions, and revision events that occurred around potential boundary positions. A boundary label *c*_*i, t*_ = 1 was assigned when a token transition corresponded to a syntactic or translation unit boundary and was supported by process level evidence. Otherwise, *c*_*i, t*_ = 0. To reduce noise in the automatically derived boundary labels, a subset of the segmented data was manually inspected by two trained annotators. The annotators evaluated whether the predicted or derived boundaries corresponded to meaningful translation units. Inter rater agreement was measured using Cohen's κ, and disagreements were resolved through discussion. The final boundary detector was evaluated on the validation and test sets using boundary precision, boundary recall, boundary F1, and WindowDiff:


$$\begin{array}{l} Precision_{b}=\frac{TP_{b}}{TP_{b}+FP_{b}},\text{ Recal}{\text{l}}_{b}=\frac{TP_{b}}{TP_{b}+FN_{b}}, \end{array}$$ (60)

$$\begin{array}{l} F1_{b}=\frac{2\text{Precisio}{\text{n}}_{b}\text{Recal}{\text{l}}_{b}}{\text{Precisio}{\text{n}}_{b}+\text{Recal}{\text{l}}_{b}}. \end{array}$$ (61)

These validation procedures ensure that the segmenter produces linguistically and cognitively meaningful boundaries rather than arbitrary attention based cuts. The segmentation results are used only as intermediate structure for segment level cognitive load estimation.

### 4.3 Baseline models

To evaluate whether the proposed model provides improvements beyond conventional regression, sequence modeling, and probabilistic baselines, we compare it with the following baseline methods. We include feature based regression models. The Ridge Regression and Support Vector Regression baselines use manually designed linguistic and behavioral features, including source sentence length, token length, word frequency, part of speech information, dependency distance, lexical diversity, pause duration, revision frequency, and completion time. These baselines evaluate whether cognitive load can be predicted from interpretable shallow features. We include non neural and probabilistic baselines. Random Forest Regression is used as a nonlinear feature based baseline. Gaussian Process Regression with an RBF kernel is included as a probabilistic regression baseline that can provide predictive uncertainty. These models serve as comparisons for both point prediction and uncertainty estimation. We include neural sequence baselines. The BiLSTM model captures sequential dependencies in the translation process. The Transformer Encoder baseline uses self attention but does not include hierarchical attention, variational constraints, or uncertainty regularization. We also include a structured state space model baseline to evaluate whether temporal cognitive load trajectories can be captured by state transition dynamics. We include hierarchical and uncertainty aware variants. The Hierarchical Attention Network baseline contains token and segment level attention but does not include the proposed variational module or uncertainty regularizer. The Transformer with MC Dropout baseline estimates uncertainty through stochastic dropout during inference. These baselines allow us to isolate whether the proposed hierarchical, variational, and uncertainty aware components improve cognitive load estimation.

### 4.4 Statistical significance testing and effect sizes

To determine whether the observed improvements are statistically reliable, we conducted paired bootstrap significance testing over the test instances. We repeatedly sampled test instances with replacement for 10,000 bootstrap iterations and computed the performance difference between the proposed model and the strongest baseline in each iteration. For point prediction metrics, the strongest baseline was the Hierarchical Attention Network. For probabilistic prediction, the strongest baseline was Transformer + MC Dropout. For each comparison, we report the mean difference, 95% confidence interval, and *p* value. The *p* value was estimated as the proportion of bootstrap samples in which the baseline outperformed or matched the proposed model. To account for multiple comparisons, Holm Bonferroni correction was applied. Effect sizes were reported using Cohen's *d*, computed from paired instance level absolute error differences:


$$\begin{array}{l} d=\frac{\bar{\text{Δ}e}}{s_{\text{Δ}e}}, \end{array}$$ (62)

where $\text{Δ}e_{i}=|y_{i}-{ŷ}_{i}^{baseline}|-|y_{i}-{ŷ}_{i}^{ours}|$, $\bar{\text{Δ}e}$ is the mean paired error reduction, and *s*_Δ*e*_ is the standard deviation of the paired error reduction. Larger positive values indicate stronger improvement of the proposed model over the baseline.

As shown in Table 6, the proposed model outperforms all baseline methods across regression and probabilistic evaluation metrics. Compared with feature based regression models, the neural sequence models achieve lower prediction errors, suggesting that contextual and temporal information is important for cognitive load estimation. However, the Transformer Encoder and BiLSTM baselines still underperform the hierarchical models, indicating that modeling token, segment, and document level structure is useful for translation task cognitive load prediction. The proposed model further improves over the Hierarchical Attention Network, reducing RMSE from 0.648 to 0.612 and MAE from 0.497 to 0.471. This suggests that the variational and uncertainty aware components provide benefits beyond hierarchical attention alone. The proposed model also achieves a lower NLL than Transformer + MC Dropout, indicating stronger probabilistic prediction and better uncertainty modeling. Table 7 reports the statistical significance testing results. The improvements of the proposed model over the strongest baseline are statistically significant after Holm Bonferroni correction. The effect sizes are in the medium range, indicating that the gains are not only statistically reliable but also practically meaningful for cognitive load estimation in translation education.

**Table 6:** Comparison with baseline models on cognitive load regression.

Model	RMSE ↓	MAE ↓	Pearson *r* ↑	Spearman ρ ↑	NLL ↓
Ridge regression	0.742	0.583	0.512	0.498	1.134
Support vector regression	0.719	0.562	0.538	0.521	1.096
Random forest regression	0.704	0.548	0.557	0.540	1.083
Gaussian process regression	0.692	0.536	0.581	0.563	1.061
BiLSTM	0.688	0.531	0.594	0.576	1.045
Transformer encoder	0.681	0.524	0.612	0.590	1.027
Structured state space model	0.659	0.506	0.641	0.623	0.972
Hierarchical attention network	0.648	0.497	0.657	0.634	0.958
Transformer + MC dropout	0.663	0.510	0.633	0.612	0.946
Ours	**0.612**	**0.471**	**0.704**	**0.686**	**0.831**

Lower RMSE, MAE, and NLL indicate better performance, while higher Pearson and Spearman correlations indicate better performance. The bolded values represent the optimal values.

**Table 7:** Statistical significance testing and effect sizes.

Metric	Compared baseline	Difference	95% CI	*p* value/Cohen's *d*
RMSE ↓	Hierarchical attention network	−0.036	[−0.052, −0.020]	*p* < 0.001, *d* = 0.58
MAE ↓	Hierarchical attention network	−0.026	[−0.039, −0.014]	*p* = 0.002, *d* = 0.52
Pearson *r* ↑	Hierarchical attention network	+0.047	[0.021, 0.073]	*p* = 0.004, *d* = 0.49
Spearman ρ ↑	Hierarchical attention network	+0.052	[0.025, 0.079]	*p* = 0.003, *d* = 0.51
NLL ↓	Transformer + MC dropout	−0.115	[−0.162, −0.068]	*p* < 0.001, *d* = 0.57

Point prediction metrics are compared with the strongest deterministic baseline, while NLL is compared with the strongest probabilistic baseline.

### 4.5 Uncertainty evaluation and calibration

Because the proposed model is designed to produce both point predictions and predictive uncertainty, we evaluate not only regression accuracy but also uncertainty calibration. For each instance *i*, the model outputs a Gaussian predictive distribution:


$$\begin{array}{l} p\left(y_{i}|x_{i}\right)=N\left(\mu_{i},\sigma_{i}^{2}\right), \end{array}$$ (63)

where μ_*i*_ denotes the predicted cognitive load value and σ_*i*_ denotes the predicted standard deviation. The standard deviation is constrained to be positive using a softplus transformation:


$$\begin{array}{l} \sigma_{i}=\text{Softplus}\left(s_{i}\right)+\epsilon , \end{array}$$ (64)

where *s*_*i*_ is the raw variance output of the model and ϵ = 10^−6^ is used for numerical stability.

We report negative log likelihood (NLL), which evaluates whether the predicted probability distribution assigns high likelihood to the observed cognitive load values:


$$\begin{array}{l} \text{NLL}=\frac{1}{N}\overset{N}{\underset{i=1}{\sum }}\left[\frac{{\left(y_{i}-\mu_{i}\right)}^{2}}{2\sigma_{i}^{2}}+\frac{1}{2}\log\left(\sigma_{i}^{2}\right)+\frac{1}{2}\log\left(2\pi \right)\right]. \end{array}$$ (65)

We evaluate calibration using prediction interval coverage. For a nominal confidence level γ, the prediction interval is defined as:


$$\begin{array}{l} I_{i}\left(\gamma \right)=\left[\mu_{i}-z_{\frac{1+\gamma }{2}}\sigma_{i},\mu_{i}+z_{\frac{1+\gamma }{2}}\sigma_{i}\right], \end{array}$$ (66)

where $z_{\frac{1+\gamma }{2}}$ is the corresponding quantile of the standard normal distribution. The prediction interval coverage probability (PICP) is calculated as:


$$\begin{array}{l} \text{PICP}\left(\gamma \right)=\frac{1}{N}\overset{N}{\underset{i=1}{\sum }}I\left(y_{i}\in I_{i}\left(\gamma \right)\right). \end{array}$$ (67)

A well calibrated model should have empirical coverage close to the nominal confidence level. Therefore, we compute expected calibration error (ECE) over a set of confidence levels Γ = {0.50, 0.60, 0.70, 0.80, 0.90, 0.95}:


$$\begin{array}{l} \text{ECE}=\frac{1}{\left|\Gamma \right|}\underset{\gamma \in \Gamma }{\sum }|\text{PICP}\left(\gamma \right)-\gamma |. \end{array}$$ (68)

We also report the mean prediction interval width (MPIW), which measures the sharpness of the predicted uncertainty interval:


$$\begin{array}{l} \text{MPIW}\left(\gamma \right)=\frac{1}{N}\overset{N}{\underset{i=1}{\sum }}2z_{\frac{1+\gamma }{2}}\sigma_{i}. \end{array}$$ (69)

To compare the proposed uncertainty aware model with non uncertainty baselines, deterministic regression models are converted into Gaussian predictors by assigning a fixed variance estimated from validation set residuals. This provides a fair reference for NLL and interval coverage while preserving the fact that these baselines do not model instance specific uncertainty. We include an MC dropout baseline, which estimates uncertainty by performing multiple stochastic forward passes at test time.

**Figure 4:**
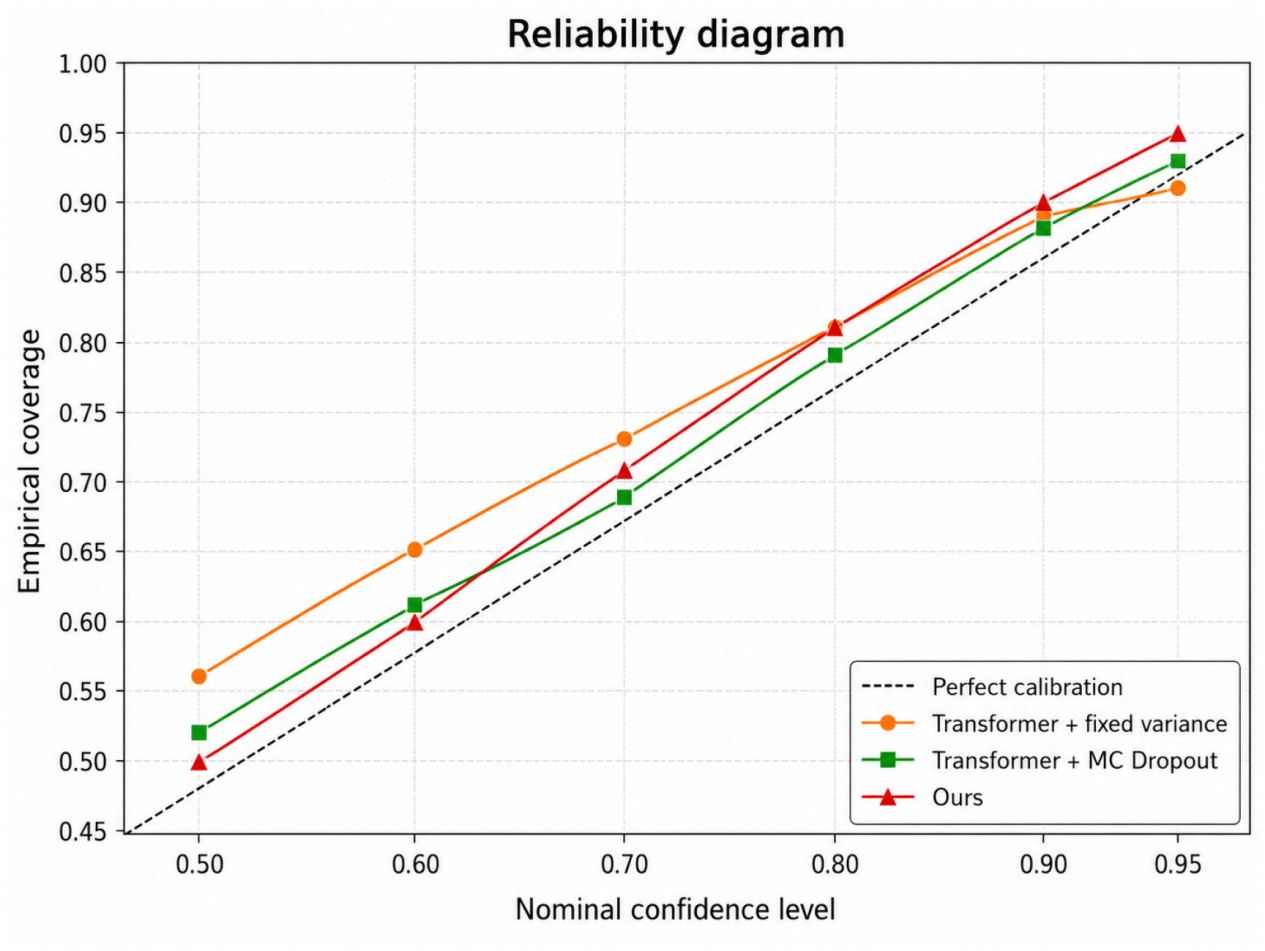
Reliability diagram for uncertainty calibration. The x axis denotes nominal confidence levels, and the y axis denotes empirical prediction interval coverage. A perfectly calibrated model should lie close to the diagonal line. The proposed model shows empirical coverage closer to the nominal confidence levels than deterministic and MC dropout baselines.

As shown in Table 8, the proposed model achieves the lowest NLL and ECE among all compared methods, indicating that it provides better calibrated probabilistic predictions. Compared with the Transformer baseline with fixed validation set variance, the proposed model reduces ECE from 0.076 to 0.026 and improves PICP@95% from 0.891 to 0.948, making the empirical coverage much closer to the nominal 95% level. Compared with MC dropout, the proposed model also obtains lower NLL and narrower prediction intervals, suggesting that its uncertainty estimates are both better calibrated and sharper. Figure 4 presents the reliability diagram of different models. The deterministic baselines with fixed variance tend to be under confident or over confident across different confidence levels because they use the same uncertainty estimate for all instances. The MC dropout baseline improves calibration by introducing stochastic predictive uncertainty, but its empirical coverage still deviates from the nominal confidence levels. In contrast, the proposed model produces instance specific predictive uncertainty and achieves coverage values closer to the diagonal reference line, indicating better calibration. These results are important for educational applications. In translation education, uncertainty estimates can help prevent overconfident instructional decisions. When the model predicts high cognitive load with high uncertainty, the system can flag the case for instructor review rather than automatically assigning a difficulty label or intervention. Therefore, uncertainty calibration provides practical value beyond improving numerical prediction performance.

**Table 8:** Uncertainty calibration comparison between the proposed model and baseline methods.

Model	RMSE ↓	NLL ↓	ECE ↓	PICP@90%	PICP@95%	MPIW@95% ↓
Ridge regression + fixed variance	0.742	1.134	0.094	0.812	0.873	2.48
Transformer + fixed variance	0.681	1.027	0.076	0.835	0.891	2.22
Transformer + MC dropout	0.663	0.946	0.049	0.879	0.926	2.05
Ours without uncertainty regularization	0.624	0.887	0.052	0.887	0.931	1.96
Ours	**0.612**	**0.831**	**0.026**	**0.902**	**0.948**	**1.84**

Lower NLL, ECE, and MPIW indicate better probabilistic prediction or sharper uncertainty estimates, while PICP should be close to the nominal confidence level. The bolded values represent the optimal values.

### 4.6 Ablation study and sensitivity analysis

To isolate the contribution of each component in the proposed framework, we conducted a set of ablation experiments. Starting from the full model, we removed one module at a time while keeping the same data split, optimizer, batch size, learning rate, and training epochs. The evaluated components include the linguistic feature encoder, adaptive temporal segmenter, hierarchical attention module, variational constraint module, and uncertainty regularizer.

The ablation results are reported in Table 9. Removing the hierarchical attention module leads to a clear performance drop, indicating that token, segment, and document level aggregation is important for modeling cognitive load at multiple levels. Removing the adaptive temporal segmenter also degrades performance, suggesting that cognitively meaningful temporal segmentation is useful for capturing local changes in translation difficulty. The removal of the variational constraint module increases both prediction error and NLL, showing that latent regularization contributes to more stable probabilistic regression. Removing the uncertainty regularizer has a relatively smaller effect on RMSE and MAE but substantially worsens NLL and calibration, indicating that this module mainly improves probabilistic reliability rather than only point prediction accuracy.

**Table 9:** Ablation study isolating the contribution of each model component.

Model variant	RMSE ↓	MAE ↓	Pearson *r* ↑	NLL ↓	ECE ↓
Full model	**0.612**	**0.471**	**0.704**	**0.831**	**0.026**
w/o linguistic features	0.634	0.489	0.676	0.879	0.034
w/o adaptive temporal segmenter	0.641	0.496	0.662	0.902	0.038
w/o hierarchical attention	0.657	0.508	0.643	0.936	0.043
w/o variational constraint module	0.646	0.501	0.654	0.921	0.041
w/o uncertainty regularizer	0.624	0.482	0.689	0.887	0.052
w/o variational module and uncertainty regularizer	0.661	0.513	0.631	0.964	0.061

Lower RMSE, MAE, NLL, and ECE indicate better performance, while higher Pearson correlation indicates better prediction quality. The bolded values represent the optimal values.

We further conducted sensitivity analyses for key hyperparameters, including the KL regularization weight λ_KL_, the smoothness weight λ_smooth_, the hierarchical consistency weight λ_hier_, the variance regularization weight λ_var_, the prior distribution used in the variational module, and the KL annealing schedule. These analyses examine whether the proposed model is robust to reasonable changes in hyperparameter settings rather than relying on a single carefully tuned configuration.

For each sensitivity experiment, only the target hyperparameter was changed, while all other settings were kept unchanged. The results show that moderate auxiliary weights produce the best trade off between point prediction and uncertainty calibration. Very small weights weaken the corresponding regularization effect, whereas overly large weights over constrain the model and reduce its ability to fit cognitive load variations. The model performs best with a standard Gaussian prior and a linear KL annealing schedule, suggesting that gradual introduction of the KL term helps stabilize variational learning.

As shown in Table 9, each proposed component contributes to the final performance. Removing hierarchical attention causes one of the largest drops in RMSE and Pearson correlation, confirming that multi level representation is important for cognitive load modeling. Removing the adaptive temporal segmenter also weakens performance, indicating that temporal boundary information helps capture local changes in processing difficulty. The variational constraint module improves both point prediction and probabilistic prediction, as reflected by lower RMSE and NLL. The uncertainty regularizer mainly improves calibration: although its removal only moderately affects RMSE, it increases ECE from 0.026 to 0.052.

The sensitivity results in Table 10 show that the model performs best with moderate auxiliary loss weights. In particular, λ_KL_ = 0.01, λ_smooth_ = 0.10, λ_hier_ = 0.20, and λ_var_ = 0.05 provide the best overall balance between prediction accuracy and uncertainty calibration. Smaller weights reduce the effect of regularization, while larger weights over constrain the model and lead to higher prediction errors.

**Table 10:** Sensitivity analysis of loss weights.

Setting	RMSE ↓	MAE ↓	NLL ↓	ECE ↓
λ_KL_ = 0.001	0.626	0.483	0.872	0.034
λ_KL_ = 0.01	**0.612**	**0.471**	**0.831**	**0.026**
λ_KL_ = 0.05	0.621	0.480	0.858	0.031
λ_KL_ = 0.10	0.639	0.493	0.901	0.040
λ_smooth_ = 0.05	0.620	0.479	0.851	0.030
λ_smooth_ = 0.10	**0.612**	**0.471**	**0.831**	**0.026**
λ_smooth_ = 0.20	0.625	0.485	0.866	0.033
λ_hier_ = 0.10	0.623	0.481	0.855	0.032
λ_hier_ = 0.20	**0.612**	**0.471**	**0.831**	**0.026**
λ_hier_ = 0.40	0.628	0.487	0.874	0.036
λ_var_ = 0.01	0.619	0.480	0.861	0.035
λ_var_ = 0.05	**0.612**	**0.471**	**0.831**	**0.026**
λ_var_ = 0.10	0.622	0.482	0.852	0.030

Each row changes one hyperparameter while keeping the remaining settings fixed. The bolded values represent the optimal values.

Table 11 shows that the standard Gaussian prior with linear KL annealing achieves the best overall results. Without KL annealing, the variational module is introduced too strongly at the beginning of training, which leads to less stable optimization. Sigmoid and cyclical annealing also produce competitive results, but they do not outperform the linear schedule in the present experiments. More complex priors, such as learnable Gaussian or mixture Gaussian priors, do not yield clear improvements, possibly because the available cognitive load data are not large enough to support more flexible latent prior structures.

**Table 11:** Sensitivity analysis of prior choices and KL annealing schedules in the variational module.

Configuration	RMSE ↓	MAE ↓	Pearson *r* ↑	NLL ↓	ECE ↓
Standard Gaussian prior, no annealing	0.627	0.486	0.678	0.873	0.036
Standard Gaussian prior, linear annealing	**0.612**	**0.471**	**0.704**	**0.831**	**0.026**
Standard Gaussian prior, sigmoid annealing	0.616	0.475	0.697	0.842	0.028
Standard Gaussian prior, cyclical annealing	0.620	0.479	0.691	0.851	0.031
Learnable Gaussian prior, linear annealing	0.618	0.476	0.695	0.846	0.029
Mixture Gaussian prior, linear annealing	0.625	0.484	0.681	0.864	0.034

The bolded values represent the optimal values.

### 4.7 Interpretability analysis and educator facing case study

To ensure that the proposed model is interpretable beyond attention visualization, we evaluated whether its high load predictions were aligned with established indicators of translation difficulty and cognitive processing demand. We examined the relationship between predicted cognitive load and linguistic, behavioral, and psycholinguistic difficulty indices, including token length, word frequency, syntactic dependency length, language model surprisal, fixation duration, pause duration, and revision density. For each segment, we computed the average predicted cognitive load and correlated it with the corresponding difficulty indicators. Pearson correlation was used to measure linear association, while Spearman correlation was used to assess rank order consistency. This analysis allows us to examine whether the model assigns higher cognitive load scores to segments that are independently known to be more difficult for learners to process. We applied SHAP analysis to the engineered linguistic and behavioral features used by the prediction head. SHAP values were computed on the validation and test sets to identify which features contributed most strongly to high cognitive load predictions. This provides a feature level explanation complementary to the hierarchical attention mechanism. While attention weights indicate which textual units receive greater model focus, SHAP values help explain which linguistic or behavioral properties drive the prediction.

sTo further demonstrate educator facing interpretability, we conducted a case analysis of high load translation segments. For each case, we report the source segment, the predicted cognitive load level, the main explanatory indicators, and the corresponding pedagogical interpretation. This analysis is intended to show how model outputs can be converted into actionable instructional feedback rather than remaining abstract numerical predictions. As shown in Table 12, segments with high predicted cognitive load often involve syntactic reordering, low frequency expressions, long dependency structures, or culturally specific phrases. These segments also tend to show longer fixation duration, longer pauses, and more revision operations. Such evidence can help instructors identify where learners require additional scaffolding, such as pre teaching difficult expressions, explaining syntactic transformations, or designing targeted revision exercises. The correlation results in Table 13 show that the model's predicted cognitive load scores are positively associated with established difficulty indicators. In particular, fixation duration, language model surprisal, pause duration, and translation error rate show relatively strong correlations with predicted cognitive load. This suggests that the model does not merely rely on opaque neural representations, but captures sources of difficulty that are consistent with psycholinguistic and translation process evidence. The SHAP results in Table 14 further show that fixation duration, surprisal, pause duration, dependency length, and revision density are among the most influential feature groups. These findings are consistent with the assumption that cognitive load in translation is affected by both source text comprehension difficulty and target text production effort. The case study in Table 12 illustrates how the model's predictions can be used in educational practice. Instead of simply reporting a high cognitive load score, the model can identify the likely sources of difficulty and support concrete instructional actions, such as vocabulary scaffolding, syntactic restructuring exercises, reformulation training, and instructor review of uncertain cases.

**Table 12:** Educator facing case study of high cognitive load translation segments.

Source segment	Predicted load	Main explanatory indicators	Pedagogical interpretation
Despite the apparent simplicity of the sentence, its implied causal relation is difficult to preserve in translation.	High	High surprisal; long dependency length; increased pause duration	The instructor may explain implicit logical relations and provide examples of causal restructuring in the target language.
The policy was eventually phased out after several rounds of institutional negotiation.	High	Low frequency phrase; long fixation duration; high revision density	The phrase phased out and the abstract institutional context may require pre teaching of policy related vocabulary and collocation practice.
Students are expected to reformulate the argument rather than translate each clause literally.	Medium high	Syntactic reordering; increased deletion and revision operations	The segment can be used to teach reformulation strategies and to discourage word by word translation.
The results were not only statistically significant but also pedagogically meaningful.	Medium	Moderate surprisal; moderate fixation duration	The instructor may highlight the distinction between statistical and educational significance to support academic translation training.

**Table 13:** Correlation between predicted cognitive load and established difficulty indicators.

Difficulty indicator	Pearson *r*	Spearman ρ
Language model surprisal	0.58	0.55
Syntactic dependency length	0.46	0.43
Token/phrase length	0.39	0.37
Inverse word frequency	0.42	0.40
Fixation duration	0.61	0.59
Pause duration	0.57	0.54
Revision density	0.49	0.47
Translation error rate	0.53	0.51

Higher positive correlations indicate stronger alignment between model predictions and known sources of translation difficulty.

**Table 14:** Top feature contributors identified by SHAP analysis.

Feature group	Mean absolute SHAP value
Fixation duration	0.184
Language model surprisal	0.162
Pause duration	0.151
Syntactic dependency length	0.137
Revision density	0.126
Inverse word frequency	0.118
Pupil dilation	0.106
Token/phrase length	0.092
Completion time	0.087

The values indicate the mean absolute SHAP value of each feature group.

## 5 Educational implications and adaptive learning applications

Although the proposed framework is computational in form, its intended contribution is educational rather than merely technical. The estimated cognitive load values can be used as diagnostic signals that help teachers and learning systems identify where, when, and why learners experience excessive processing demands during translation. In this sense, the model provides a bridge between cognitive load theory and actionable instructional design in English translation education.

### 5.1 Instructional diagnosis

The first educational use of the proposed model is instructional diagnosis. Because the model estimates cognitive load at token, segment, and document levels, it can help identify linguistic units that impose unusually high processing demands on learners. At the token level, high predicted load may indicate unfamiliar vocabulary, low frequency expressions, terminology, or culturally specific items. At the segment level, high load may reflect syntactic restructuring, long distance dependencies, ambiguous reference, or dense information packaging. At the document level, high load may indicate that the overall task exceeds the learner's current proficiency or working memory capacity. Such diagnostic information can help instructors move beyond final translation scores and examine the process level sources of difficulty. For example, two learners may obtain similar translation scores but experience different cognitive load patterns: one may struggle mainly with lexical access, whereas another may struggle with sentence restructuring or discourse level coherence. By locating these differences, the model can support more individualized instructional decisions.

### 5.2 Learner feedback

The second educational use is learner feedback. Traditional feedback in translation education often focuses on product level errors, such as mistranslation, grammar problems, or inappropriate word choice. The proposed framework can complement such feedback by providing process oriented information about cognitive effort. Instead of only telling learners which translation is incorrect, the system can indicate which parts of the source text are likely to have caused excessive cognitive load and explain possible reasons for the difficulty. For instance, if a learner shows high estimated cognitive load on a segment containing an embedded clause, the feedback can prompt the learner to first identify the clause boundary, then determine the main predicate, and reorganize the information in the target language. If high load is associated with terminology, the feedback can recommend glossary review or parallel text comparison. If high load is associated with repeated revisions, the feedback can encourage planning before drafting. In this way, model outputs can be converted into metacognitive feedback that helps learners understand their own translation process.

### 5.3 Curriculum development

The third use concerns curriculum development. Aggregated cognitive load estimates across learners and tasks can help instructors evaluate whether the sequence of translation exercises is appropriately graded. If many learners show high cognitive load on a particular type of structure, such as passive constructions, nominalizations, idioms, or culturally embedded expressions, the curriculum may need additional scaffolding before assigning more complex translation tasks. The model can also support the design of progressive translation materials. Tasks with lower estimated cognitive load can be used for initial schema construction, while tasks with moderate load can be used for guided practice. Tasks with consistently excessive load may be postponed, simplified, or accompanied by additional instructional support. This use is consistent with cognitive load theory, which emphasizes the need to manage intrinsic load, reduce unnecessary extraneous load, and promote productive germane processing during learning.

### 5.4 Intervention strategies and adaptive learning

The fourth use is adaptive intervention. Because the model produces continuous cognitive load estimates, it can be integrated into adaptive learning systems that adjust instructional support according to learners' current needs. When the estimated load is low, the system may reduce scaffolding and encourage independent translation. When the estimated load is moderate, the system may provide targeted hints, examples, or contrastive explanations. When the estimated load is excessively high, the system may split the task into smaller units, provide vocabulary support, highlight syntactic structure, or recommend prerequisite practice. A possible intervention workflow is as follows. The learner completes a translation task in a digital learning environment. The model estimates cognitive load at multiple linguistic levels. The system identifies high load tokens or segments and links them to possible instructional causes, such as lexical unfamiliarity, syntactic complexity, or discourse ambiguity. The system provides adaptive feedback or recommends follow up exercises. The instructor can review aggregated learner level and class level reports to adjust teaching plans. These applications demonstrate that the proposed model is not intended to replace teachers' professional judgment. Rather, it provides additional evidence that can support instructional decision making. By connecting cognitive load estimation with diagnosis, feedback, curriculum design, and adaptive intervention, the framework contributes to educational psychology by showing how computational modeling can be used to make learners' cognitive effort more visible and pedagogically actionable.

## 6 Conclusions and future work

In this study, we proposed a framework for Cross Linguistic Cognitive Load Estimation in English Translation Education. The framework integrates hierarchical attention, variational constraint optimization, temporal segmentation, and uncertainty aware prediction to estimate continuous cognitive load proxies across multiple linguistic levels. Beyond predictive modeling, the revised study emphasizes the educational role of cognitive load estimation: the model outputs can support instructional diagnosis, learner feedback, curriculum development, and adaptive intervention. In this way, the framework connects computational modeling with cognitive load theory and practical instructional design.

Despite the promising results, the study has two notable limitations. The framework relies heavily on pre defined linguistic features and domain specific constraints, which may limit its generalizability to other cross linguistic contexts or educational domains. Future research could explore more generalized feature extraction techniques or unsupervised learning approaches to broaden the applicability of the model. While the hierarchical attention mechanism effectively captures multi level dependencies, its computational complexity remains a challenge, particularly for large scale datasets. Optimizing the model's efficiency or exploring lightweight alternatives could address this issue. This study establishes a foundation for the development of adaptive tools in translation education. Future research should aim to improve scalability and generalizability to enhance the impact of these tools across various linguistic and educational contexts.

## Data Availability

The original contributions presented in the study are included in the article/supplementary material, further inquiries can be directed to the corresponding author.

## References

[B1] Alves F. Albir A. H. (2017). “Evolution, challenges, and perspectives for research on cognitive aspects of translation,” in The Handbook of Translation and Cognition, 535–554. doi: 10.1002/9781119241485.ch29

[B2] Alves F. Jakobsen A. L. (2021). The Routledge Handbook of Translation and Cognition. London: Routledge. doi: 10.4324/9781315178127

[B3] Carl M. (2012). Translog-ii: A program for recording user activity data for empirical translation process research. doi: 10.63317/4yt2527b3dza https://research.cbs.dk/files/58900336/Michael_Carl_2012.pdf

[B4] Carl M. Kay M. (2011). Gazing and typing activities during translation: a comparative study of translation units of professional and student translators. Meta 56, 952–975. doi: 10.7202/1011262ar

[B5] Dragsted B. (2005). Segmentation in translation: differences across levels of expertise and difficulty. Target. Int. J. Transl. Stud. 17, 49–70. doi: 10.1075/target.17.1.04dra

[B6] Hart S. G. Staveland L. E. (1988). “Development of nasa-tlx (task load index): results of empirical and theoretical research,” in Advances in Psychology (Elsevier), 139–183. doi: 10.1016/S0166-4115(08)62386-9

[B7] Hvelplund K. T. (2014). Eye tracking and the translation process: Reflections on the analysis and interpretation of eye-tracking data. Available online at: https://rua.ua.es/dspace/bitstream/10045/43726/1/MonTI_2014_Special_Issue_07.pdf

[B8] Jakobsen A. L. (2002). Translation drafting by professional translators and by translation students. Available online at: https://www.academia.edu/download/86898592/20122.pdf

[B9] Leppink J. Paas F. Van der Vleuten C. P. Van Gog T. Van Merriënboer J. J. (2013). Development of an instrument for measuring different types of cognitive load. Behav. Res. Methods 45, 1058–1072. doi: 10.3758/s13428-013-0334-1 23572251

[B10] O'brien S. (2006). Pauses as indicators of cognitive effort in post-editing machine translation output. Across Lang. Cult. 7, 1–21. doi: 10.1556/Acr.7.2006.1.1

[B11] Paas F. Tuovinen J. E. Tabbers H. Van Gerven P. W. (2016). “Cognitive load measurement as a means to advance cognitive load theory,” in Cognitive Load Theory (Routledge), 63–71. doi: 10.4324/9780203764770

[B12] Paas F. G. (1992). Training strategies for attaining transfer of problem-solving skill in statistics: a cognitive-load approach. J. Educ. Psychol. 84:429. doi: 10.1037/0022-0663.84.4.429

[B13] Schaeffer M. Carl M. (2013). Shared representations and the translation process: a recursive model. Transl. Interpr. Stud. 8, 169–190. doi: 10.1075/tis.8.2.03sch

[B14] Sharon O. (2007). Eye-tracking and translation memory matches. Perspectives 14, 185–205. doi: 10.1080/09076760708669037

[B15] Sweller J. (1988). Cognitive load during problem solving: effects on learning. Cogn. Sci. 12, 257–285. doi: 10.1207/s15516709cog1202_4

[B16] Sweller J. Van Merrienboer J. J. Paas F. G. (1998). Cognitive architecture and instructional design. Educ. Psychol. Rev. 10, 251–296. doi: 10.1023/A:1022193728205

[B17] Van Merrienboer J. J. Sweller J. (2005). Cognitive load theory and complex learning: recent developments and future directions. Educ. Psychol. Rev. 17, 147–177. doi: 10.1007/s10648-005-3951-0

[B18] Xiao K. Martín R. M. (2020). Cognitive translation studies: models and methods at the cutting edge. Linguist. Antverp. Series-Themes Transl. Stud. 19:593. doi: 10.52034/lanstts.v19i0.593

[B19] Yang Z. Yang D. Dyer C. He X. Smola A. Hovy E. (2016). “Hierarchical attention networks for document classification,” in Proceedings of the 2016 Conference of the North American Chapter of the Association for Computational Linguistics: Human Language Technologies, 1480–1489. doi: 10.18653/v1/N16-1174

